# Supplementary material for: Is insomnia a risk factor for new-onset asthma? A population-based study in Taiwan
Source: BMJ Open. 2017 Nov 28;7(11):e018714. doi: 10.1136/bmjopen-2017-018714 (PMC5719310; doi:10.1136/bmjopen-2017-018714)
Supplement: Author's manuscript [file bmjopen-2017-018714.draft_revisions.pdf]

BMJ Open is committed to open peer review. As part of this commitment we make the peer review history of every article we publish publicly available.

When an article is published we post the peer reviewers' comments and the authors' responses online. We also post the versions of the paper that were used during peer review. These are the versions that the peer review comments apply to.

The versions of the paper that follow are the versions that were submitted during the peer review process. They are not the versions of record or the final published versions. They should not be cited or distributed as the published version of this manuscript.

BMJ Open is an open access journal and the full, final, typeset and author-corrected version of record of the manuscript is available on our site with no access controls, subscription charges or pay-per-view fees (http://bmjopen.bmj.com).

If you have any questions on BMJ Open's open peer review process please email editorial.bmjopen@bmj.com

BMJ Open

Is Insomnia a Risk Factor for New-Onset Asthma? A Population-Based Study

Journal:	*BMJ Open*
Manuscript ID	bmjopen-2017-018714
Article Type:	Research
Date Submitted by the Author:	17-Jul-2017
Complete List of Authors:	Lin, Yu-Chieh; Chi Mei Medical Center, Family Medicine<br>Lai, Chih-Cheng; Chi Mei Medical Center, Liouying, Intensive Care Medicine<br>Chien, Chih-Chiang ; Chi Mei Medical Center, Internal Medicine<br>Chen, Chin-ming; Intensive Care Medicine<br>Chiang, Shyh-Ren; Chi Mei Medical Center, Internal Medicine<br>Ho, Chung-Han; Chi Mei Medical Center, Medical Research<br>Weng, Shih-Feng; Chi Mei Medical Center, Departmetn of Medical Research<br>Cheng, Kuo-Chen; Chi Mei Medical Center, Internal Medicine
**Primary Subject Heading**:	Epidemiology
Secondary Subject Heading:	Respiratory medicine
Keywords:	EPIDEMIOLOGY, Asthma < THORACIC MEDICINE, SLEEP MEDICINE

SCHOLARONE™
Manuscripts

For peer review only

Research Paper

Is Insomnia a Risk Factor for New-Onset Asthma? A Population-Based Study

Running title: Insomnia and Risk of Asthma

Yu-Chieh Lin, MD[1]; Chih-Cheng Lai, MD[2]*; Chih-Chiang Chien, MD[3]; Chin-Ming Chen, MD[4,5]; Shyh-Ren Chiang, MD[3,5]; Chung-Han Ho, PhD[6,7]; Shih-Feng Weng, PhD[8]; Kuo-Chen Cheng, MD[3,9]*

Departments of [1]Family Medicine, [3]Internal Medicine, [4]Intensive Care Medicine, and [6]Medical Research, Chi Mei Medical Center, Tainan, Taiwan;

[2]Department of Intensive Care Medicine, Chi Mei Medical Center, Liouying, Taiwan;

Departments of [5]Recreation and Healthcare Management, and [7]Pharmacy, Chia Nan University of Pharmacy & Science, Tainan, Taiwan; [8]Department of Healthcare Administration and Medical Informatics, Kaohsiung Medical University, Kaohsiung, Taiwan; and [9]Department of Safety, Health, and Environmental Engineering, Chung Hwa University of Medical Technology, Tainan, Taiwan

*The first and second authors contributed equally to this work.

Correspondence: Kuo-Chen Cheng, MD, Department of Internal Medicine, Chi Mei Medical Center, Tainan, Taiwan. 901 Zhonghua Road, Yong Kang District, Tainan City 710, Taiwan

Tel: 886-6-281-2811 Ext 57111; Fax: 886-6-282-8928; E-mail: kcg.cheng@gmail.com

For peer review only

Abstract

Objectives: To determine whether insomnia at baseline is a risk factor for new-onset asthma.

Design: A cohort study

Setting: Taiwan National Health Insurance Research Database.

Participants: We recruited 48,871 patients with insomnia (Insomnia group) newly diagnosed between 2002 and 2007, and 97,742 matched controls without insomnia (Control group) from Taiwan's Longitudinal Health Insurance Database 2000. All of the patients were followed up for 4 years to see whether new-onset asthma developed. Patients with previous asthma or insomnia were excluded.

Major outcome measurement: The incidence rate ratios (IRRs) of asthma were estimated by the Poisson regression, and Cox proportional hazard regression was used to calculate the risk of asthma between the two groups.

Results: After a 4-year follow-up, 424 patients in the Insomnia group and 409 in the Control group developed asthma. The incidence rate of asthma was significantly higher in the insomnia group (22.01 vs. 10.57 per 10,000 person-years). Patients with insomnia have a higher risk of developing new-onset asthma during the 4-year follow-up (hazard ratio [HR]: 2.08, 95% CI: 1.82-2.39). The difference remained significant after adjustment (adjusted HR: 1.89, 95% CI: 1.64-2.17).

Conclusions: This large population-based study suggests that insomnia at baseline is a risk factor for developing asthma.

Keywords: Insomnia, asthma, comorbid, medical disorders, risk.

For peer review only

Strengths and Limitations of this study

1. The major strengths of our study are its prospective cohort design and that we used the LHID2000, a subset of Taiwan's National Health Insurance Research Database (NHIRD), which reflects the real-world situation in Taiwan and is more generalizable than are hospital-based or city-based databases.
2. The NHIRD database does not include some possible confounding factors, such as smoking habits, house dust mite exposure, etc..
3. The NHIRD database NHIRD does not contain patient information before 1996, some of our patients might have been misclassified if they were diagnosed with asthma or insomnia before that year.

For peer review only

Introduction

Insomnia is a sleep disorder that makes falling asleep or staying asleep difficult, or prevents people who awaken early in the morning from returning to sleep at least three nights per week for at least three months, despite an adequate opportunity for sleep.[1] The prevalence of insomnia is about 25% in Taiwanese adults[2]; and other countries report prevalences ranging from 9% to 50%.[3-5] Insomnia has long been linked with multiple physical conditions, allergies, cancer, hypertension, diabetes, migraine, headache, osteoporosis, fibromyalgia, rheumatoid arthritis, arthrosis, musculoskeletal disorders, and obesity.[4] Moreover, impaired sleep is related to developing anxiety and depression.[6-8] It also burdens the public health sector, reduces quality of life, and leads to more traffic accidents.[9-15]

Asthma is a heterogeneous disease characterized by recurrent attacks of breathlessness and wheezing caused by chronic airway inflammation.[16] Patients with severe asthma may have the problems of insufficient sleep, poor sleep hygiene, and clinically significant insomnia.[17,18] Although it is well known that insomnia is more prevalent in patients with asthma,[4,19] Sivertsen et al.[20] reported that the association might be bidirectional. They did not, however, focus on a single disease, and whether a respondent had asthma was based only on answers from a personal questionnaire rather than on a professional diagnosis. Nor did the study adjust for confounding factors. Therefore, we did a prospective cohort study based on clinical diagnoses to determine whether insomnia is a risk factor for new-onset asthma.

Materials and Methods

Study design and cohorts

Taiwan launched a single-payer National Health Insurance (NHI) program on March 1, 1995. The NHI databases, one of the largest and most complete population-based datasets in

For peer review only

the world, include medical claims information on almost the entire population in Taiwan (> 98% in 2009). The data used in this study were taken from the Longitudinal Health Insurance Database 2000 (LHID2000), which contains all claims data from 1996 to 2011 of 1 million (ca. 5% of all enrollees) representative beneficiaries randomly selected from Taiwan's National Health Insurance Research Database (NHIRD) in 2000. The LHID2000 provides encrypted patient identification numbers, sex, date of birth, dates of admission and discharge, the International Classification of Diseases-9th Revision-Clinical Modification (ICD-9-CM) codes of diagnoses and procedures, details of prescriptions, and expenditure amounts for all outpatient and inpatient medical benefit claims. The institutional review board of Chi Mei Medical Center approved the study and waived the requirement of informed consent because the datasets analyzed contained only deidentified patient information.

Participants

We did a prospective cohort study with two study groups: the Insomnia group (patients with newly diagnosed insomnia from 2002 to 2007) and an age-, sex-, and index-date-matched Control group (patients without insomnia). Patients in the Insomnia group (ICD-9-CM codes: 307.41, 307.42, 780.50, 780.52) were diagnosed with insomnia during at least one inpatient hospitalization or more than 3 times during outpatient clinic visits within one year. Patients diagnosed with asthma (ICD-9-CM code: 493) before insomnia were excluded.

Each patient with insomnia was age-, sex-, and index-date-matched to two patients without insomnia. The index dates for the Insomnia group patients were the dates of their first registration. Those index dates were used to create index dates for each Control group patient. To investigate the risks of developing asthma during the follow-up period, we tracked each participant no more than 4 years from their index date until new-onset asthma, death, or the end of the 4-year follow-up. The asthma was also defended as patients with asthma diagnosis

For peer review only

at least one inpatient hospitalization or more than 3 times during outpatient clinic visits within one year.

Definition of comorbidities

The diagnoses of different physical and mental conditions were based on ICD-9-CM codes during at least one inpatient hospitalization or more than 3 times during outpatient clinic visits within one year before index date. Comorbidities were as follow: hypertension (401-405, 362.11, 437.2), depression (311, 296.2, 296.3, 300.4), anxiety (293.84, 300.0x, 300.10, 300.2x, 300.5, 309.21), allergic rhinitis (477.x), urticaria (708.x), atopic dermatitis (691.x), bronchiolitis (466.11, 466.19, 079.6), sleep apnea (327.23, 780.51, 780.53, 780.57), cardiovascular disease (stroke and coronary heart disease) (430-438, 410-414).

Statistical analysis

Pearson's χ^2 test was used to compare differences in the baseline characteristics and comorbid medical disorders between the Insomnia and Control cohorts. The incidence rate was calculated as the number of asthma cases during the follow-up, divided by the total person-years for each group. Poisson regression was used to estimate the incidence rate ratios (IRRs) and 95% confidence intervals (CIs) of asthma between the two groups. A Kaplan-Meier analysis of the cumulative incidence rates of asthma was plotted to describe the proportion of patients with asthma, and the log-rank test was used to analyze the differences between the two cohorts. In addition, Cox proportional hazard regression was used to calculate the risk of asthma between patients with and without insomnia during the follow-up period. SAS 9.4 (SAS Institute, Inc., Cary, NC, USA) was used for all statistical analyses. Significance was set at $P < 0.05$ (two-sided).

Results

The Insomnia group contained 48,871 patients and the Control group contained 97,742

For peer review only

(Table 1). Insomnia was more common in women (59.96%) and in the middle-aged group (35-49 years: 33.29%). Insomnia group patients were more likely to have comorbidities, such as hypertension (HTN) (21.72% vs. 15.12%), anxiety/depression (10.15% vs. 1.69%), allergic rhinitis (3.56% vs. 1.60%), urticaria (2.37% vs. 0.97%), atopic dermatitis (0.51% vs. 0.31%), sleep apnea (0.18% vs. 0.02%), and cardiovascular disease (CVD) (10.10% vs. 6.33%) than were Control group patients. The Insomnia group tended to have lower incomes than did the Control group.

Table 1. Baseline demographic characteristics of patients in the Insomnia and Control groups

	Insomnia (n = 48,871) n (%)	Control (n = 97,742) n (%)	$P^{\dagger}$
**Age Group (years)**			
< 34	9515 (19.47)	19,031 (19.47)	1.00
35-49	16,269 (33.29)	32,541 (33.29)	
50-64	13,611 (27.85)	27,221 (27.85)	
≥ 65	9476 (19.39)	18,949 (19.39)	
**Sex**			
Male	19,569 (40.04)	39,138 (40.04)	1.00
Female	29,302 (59.96)	58,604 (59.96)	
**Comorbidity**			
Hypertension	10,616 (21.72)	14,781 (15.12)	< 0.001
Anxiety/Depression	4961 (10.15)	1649 (1.69)	< 0.001
Allergic rhinitis	1742 (3.56)	1561 (1.60)	< 0.001
Urticaria	1158 (2.37)	949 (0.97)	< 0.001
Atopic dermatitis	247 (0.51)	299 (0.31)	< 0.001
Bronchiolitis	57 (0.12)	130 (0.13)	0.44
Sleep apnea	87 (0.18)	21 (0.02)	< 0.001
Cardiovascular disease	4936 (10.10)	6187 (6.33)	< 0.001
**Geographic Region**			
North	22,674 (46.40)	46,019 (47.08)	< 0.001
Central	11,144 (22.80)	17,352 (17.75)	
South	13,803 (28.24)	32,264 (33.01)	
East	1250 (2.56)	2107 (2.16)	
**SES (monthly insurable wage in NT$)***			
< 20,000	36,415 (74.51)	69,456 (71.06)	< 0.001
20,000-40,000	8370 (17.13)	18,507 (18.93)	
> 40,000	4086 (8.36)	9779 (10.00)	

SES = socioeconomic status; NT$ = New Taiwan dollar.
* US$1 NT$30.
† *P* determined using χ^2 tests

For peer review only

After a 4-year follow-up, 424 patients in the Insomnia group and 409 patients in the Control group had developed asthma. The incidence rate of asthma was significantly higher in the Insomnia group (22.01 vs. 10.57 per 10,000 person-years; IRR: 2.08 (95% CI: 1.82-2.39; P < 0.001) (Table 2). Patients with insomnia had a higher probability of developing asthma during the 4-year follow-up (HR: 2.08; 95% CI: 1.82-2.39). This difference was still significant after adjustment (adjusted (A)HR: 1.89; 95% CI: 1.64-2.17) (Table 3). Advanced age was also related to a higher risk for developing asthma (≥ 65 years: AHR: 8.13; 95% CI: 5.89-11.23). Patients with higher incomes were less likely to develop asthma (New Taiwan dollars (NT$) > 40,000: AHR = 0.62; 95% CI: 0.44-0.89). People living in eastern Taiwan were more likely to have new-onset asthma then those living in other regions of Taiwan (AHR = 2.01; 95% CI: 1.44-2.81). The subgroup analysis showed that insomnia was associated with a greater risk of asthma in patients with HTN and CVD (Table 4). A Kaplan-Meier survival curve shows that the Insomnia group had a higher cumulative incidence rate of asthma than did the Control group (P < 0.001) (Figure 1).

For peer review only

Figure 1. The probability of asthma with year between insomnia and control group

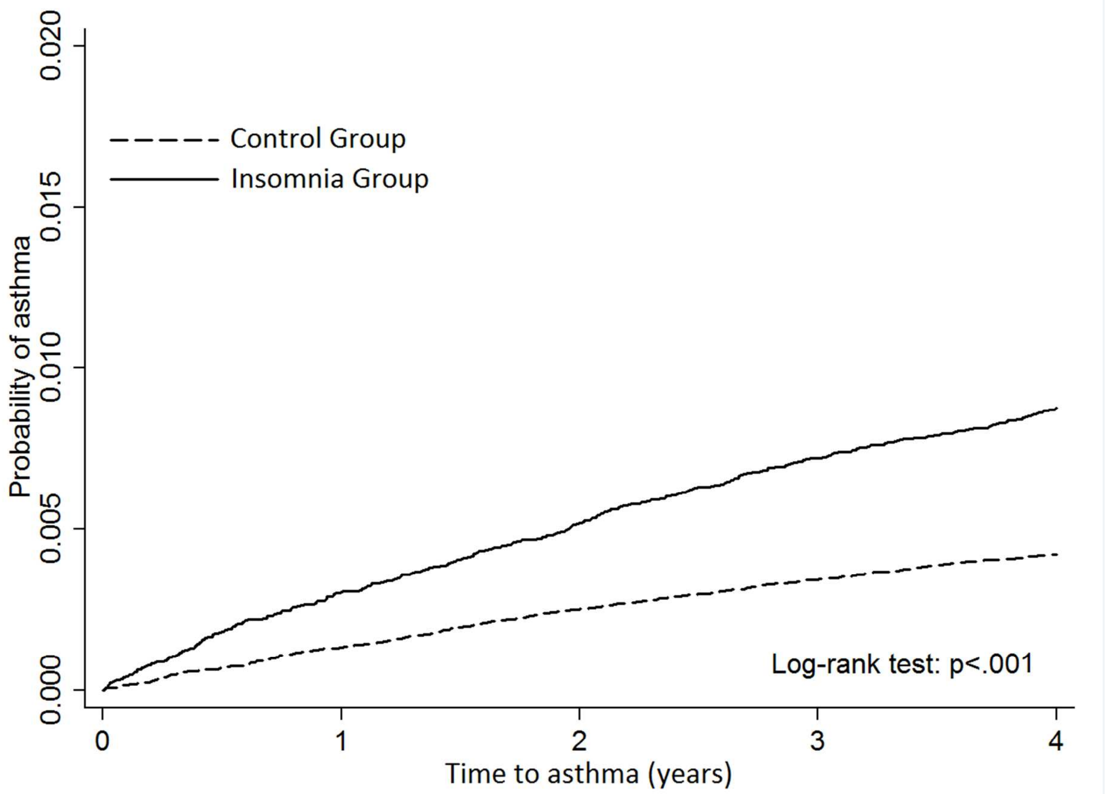

For peer review only - http://bmjopen.bmj.com/site/about/guidelines.xhtml

Table 2. Risks of asthma in the Insomnia and Control groups

Characteristics	Insomnia				Control				IRR (95% CI)	$P^{\dagger}$
	No.	Asthma	PY	IR*	No.	Asthma	PY	IR*		
**All**	48871	424	192623.41	22.01	97742	409	387095.85	10.57	2.08 (1.82-2.39)	< 0.001
**Age Group (years)**										
< 34	9515	27	37931.78	7.12	19031	17	76049.26	2.24	3.18 (1.74-5.84)	< 0.001
35-49	16269	82	64569.83	12.70	32541	32	129858.42	2.46	5.15 (3.43-7.75)	< 0.001
50-64	13611	85	53788.73	15.80	27221	83	108051.32	7.68	2.06 (1.52-2.78)	< 0.001
≥ 65	9476	230	36333.07	63.30	18949	277	73136.86	37.87	1.67 (1.40-1.99)	< 0.001
**Sex**										
Male	19569	180	76611.01	23.50	39138	179	154370.93	11.60	2.03 (1.65-2.49)	< 0.001
Female	29302	244	116012.41	21.03	58604	230	232724.93	9.88	2.13 (1.78-2.55)	< 0.001
**Comorbidity**										
Hypertension	10616	180	41264.43	43.62	14781	167	57535.99	29.03	1.50 (1.22-1.86)	< 0.001
Anxiety/Depression	4961	59	19475.68	30.29	1649	15	6417.10	23.38	1.30 (0.74-2.28)	0.37
Allergic rhinitis	1742	19	6881.46	27.61	1561	8	6157.11	12.99	2.13 (0.93-4.85)	0.07
Urticaria	949	6	3719.76	16.13	1158	6	4556.88	13.17	1.23 (0.40-3.80)	0.73
Atopic dermatitis	247	1	973.35	10.27	299	4	1152.70	34.70	0.30 (0.03-2.65)	0.28
Bronchiolitis	57	1	227.06	44.04	130	1	515.46	19.40	2.27 (0.14-6.29)	0.56
Sleep apnea	87	1	344.54	29.02	21	1	80.21	124.70	0.23 (0.01-3.72)	0.30
CVD	4936	117	18838.57	62.11	6187	114	23618.24	48.27	1.29 (0.99-1.67)	0.06

PY = person-years; IR = incidence rate; IRR = incidence rate ratio; CI = confidence interval; CVD = cardiovascular disease.

* IR: per 10,000 person-years.

† P-values were determined using Poisson regression models.

For peer review only

Table 3. Cox proportional hazard regressions for the development of asthma during the follow-up period

Variable	Crude HR (95% CI)	Adjusted HR (95% CI)[†]
**Insomnia**		
No	Ref	Ref
Yes	2.08 (1.82-2.39)	1.89 (1.64-2.17)
**Age Group**		
< 34	Ref	Ref
35-49	1.52 (1.07-2.15)	1.57 (1.11-2.22)
50-64	2.69 (1.93-3.75)	2.45 (1.75-3.43)
≥ 65	11.95 (8.78-16.26)	8.13 (5.89-11.23)
**Sex**		
Male	Ref	Ref
Female	0.88 (0.76-1.00)	1.00 (0.87-1.15)
**Comorbidity**		
Hypertension	3.47 (3.02-3.98)	1.24 (1.06-1.45)
Anxiety/Depression	2.08 (1.64-2.65)	1.19 (0.93-1.53)
Allergic rhinitis	1.46 (0.99-2.14)	1.33 (0.90-1.96)
Urticaria	1.01 (0.57-1.78)	0.81 (0.46-1.44)
Atopic dermatitis	1.64 (0.68-3.95)	1.15 (0.48-2.78)
Bronchiolitis	1.88 (0.47-7.52)	2.09 (0.52-8.38)
Sleep apnea	3.29 (0.82-13.17)	2.82 (0.70-11.37)
Cardiovascular disease	4.84 (4.16-5.63)	1.80 (1.52-2.14)
**Geographic Region**		
North	Ref	Ref
Central	1.29 (1.08-1.55)	1.26 (1.05-1.51)
South	1.28 (1.09-1.50)	1.18 (1.00-1.38)
East	2.45 (1.68-3.29)	2.01 (1.44-2.81)
**SES (monthly insurable wage in NT$)**[*]		
< 20,000	Ref	Ref
20,000-40,000	0.27 (0.21-0.36)	0.53 (0.40-0.71)
> 40,000	0.35 (0.25-0.49)	0.62 (0.44-0.89)

HR = hazard ratio; CI = confidence interval; Ref = reference value; SES = socioeconomic status.

[†] Parameters were adjusted for all covariates included in the model.

[*] US$1 NT$30.

For peer review only

Table 4. Stratified analysis for patients with hypertension or cardiovascular disease

	Patients with HTN (n = 25397) AHR (95% CI)	*P*	Patients with CVD (n = 11123) AHR (95% CI)	*P*
**Control**	1.00 (ref.)		1.00 (ref.)	
**Insomnia**	1.59 (1.28-1.97)	< 0.001	1.38 (1.06-1.79)	0.02

HTN = hypertension; CVD = cardiovascular disease; AHR = adjusted hazard ratio; CI = confidence interval; ref. = reference value.

For peer review only

Discussion

This is the largest cohort study focused on the association between insomnia and new-onset asthma, and the first one on an Asian population. We found that insomnia patients who sought medical assistance had a significant risk for developing asthma within 4 years of asking for treatment. In this study, the incidence of asthma in control group was 10.57 per 10,000 person-years. This finding is similar to the most recent epidemiology study[21] in Taiwan that the incidence of asthma was 9.8 per 10,000 person-year. In contrast, the insomnia group had significant higher incidence of asthma – 22.01 per 10,000 person-year than the control group and the general population in previous study.[21] Several studies have reported linkages between insomnia and chronic illnesses, including asthma. A cross-sectional study[19] of 3283 adults showed a higher prevalence of insomnia in those with asthma (AOR 1.6; 95% CI: 1.3-2.0). A 5-year prospective study[22] of 2316 middle-aged adults reported that patients with insomnia at baseline had a higher incidence of asthma (AOR = 17.9; 95% CI: 2.28-140) than did patients without insomnia. But no causal relationship between insomnia and asthma was supported in these two studies. Unlike those two studies, our study excluded patients who had previously been diagnosed with asthma, and we followed our patients for 4 years, which provided evidence that insomnia is a risk factor for developing asthma.

Sivertsen et al.[20] reported that insomnia was a significant risk factor for incidence of

asthma in an 11-year large population-based prospective cohort study consisted of 24715 participants (OR = 1.47; 95% CI: 1.16-1.86). However, they used questionnaires to define insomnia and provided no information about the severity or duration of their participants' poor sleep. The diagnoses of asthma and comorbidities were based on self-reports instead of physician-reported diagnoses. Their sample size was smaller than was ours, and the confounding factors adjusted for in their analysis did not include specific asthma or insomnia-related comorbidities such as atopy. In contrast, we used ICD-9 CM codes from Taiwan's LHID2000, which indicated the patients' physician-diagnosed illnesses, including insomnia, asthma, and all comorbidities. Besides, we believed that the insomniacs in our cohort suffered from more severe sleep-related symptoms that needed medical assistance. Our findings were statistically significant after our analyses had been fully adjusted for the most common confounding factors related to asthma and insomnia.

Although our findings and those of other studies[17,18,20] indicate a significant association between insomnia and asthma, the pathophysiology of insomnia-related asthma is still unclear. Nonetheless, the common inflammatory pathway between insomnia and asthma should be considered, and several mechanisms have been proposed to explain the potential relationship between insomnia and asthma. First, poor sleep increases Interleukin-6 (IL-6) production, and this response lasts until daytime.[23,24] Patients with stable and with acute asthma had significantly higher IL-6 production levels in serum, sputum, and bronchoalveolar lavage fluid than did healthy controls.[25,26] IL-6 production in the airway promotes allergic airway inflammation in mice. In contrast, using IL-6 knockout mice in the same model showed significantly less mucus secretion.[27,28] Therefore, insomnia might contribute to inducing IL-6 production and that might exacerbate airway hypersensitivity. Second, NF-κB can be induced by sleep loss.[29] Prolonged airway epithelial NF-κB activation has been reported in patients with asthma. Even temporal NF-κB activation in the airway epithelium is sufficient to induce

For peer review only

airway hyperresponsiveness in mice.[30] Thus, the higher incidence of asthma in insomnia patients might be the result of temporal or persistent NF-κB activation induced by sleep loss. Third, insomnia is related to a reduction in interferon (IFN)-γ.[31] IFN-γ production, which inhibits airway epithelial inflammation, is lower in asthma patients than in healthy controls.[32,33] These studies suggest that IFN-γ plays a significant role between insomnia and asthma.

The strengths of our study are its prospective cohort design and that we used the LHID2000, a subset of Taiwan's National Health Insurance Research Database (NHIRD), which contains physician-provided clinical diagnoses instead of illnesses self-reported by patients. The LHID2000 reflects the real-world situation in Taiwan and is more generalizable than are hospital-based or city-based databases. The large sample size and long-term follow-up also provide considerable statistical power.

Our study also has some limitations. The NHIRD does not include results of pulmonary function tests or blood tests for inflammatory cytokines, severity levels or the actual duration of insomnia, some important confounding factors, such as exposure to house dust mites, smoking habits, family history, or medical compliance. In addition, the NHIRD did not have records regarding body weight or BMI. Therefore, we cannot investigate the role of obesity which is supposed to be associated with asthma and insomnia in this study. Finally, because the NHIRD does not contain patient information before 1996, some of our patients might have been misclassified if they were diagnosed with asthma or insomnia before that year.

Conclusion

We found that insomnia patients who required medical assistance had a higher risk for developing new-onset asthma. Proper treatments for insomnia patients might help prevent the progress of airway inflammation. Additional study is needed to identify the actual mechanism

For peer review only

that connects insomnia and asthma.

Contributors:

YCL, CCL, and KCC designed the study, interpreted the data and drafted, and revised the article. CCC, CMC, and SRC contributed to interpreting the data and revising the article. CHH and SFW contributed to the statistical analysis. KCC critically reviewed and revised the article. All of the authors read and agreed with the final version of the manuscript.

Funding: This research received no specific grant from any funding agency in the public, commercial or not-for-profit sectors.

Competing interests: There are no competing interests

Data sharing statement: The data on the study population that were obtained from the NHIRD (https://nhird.nhri.org.tw/en/) are maintained in the NHRI (http://nhird.nhri.org.tw/). The NHIRD is limited for research purposes only. Applicants must follow the Computer-Processed Personal Data Protection Law (http://www.winklerpartners.com/?p=987) and related regulations of National Health Insurance Administration. All applications are reviewed for approval of data release. Interested researchers may submit queries related to data access to nhird@nhri.org.tw

For peer review only

References

1. American Psychiatric Association. Diagnostic and statistical manual of mental disorders, fifth edition. Arlington, VA: Author, 2013.

2. Kao CC, Huang CJ, Wang MY, Tsai PS. Insomnia: prevalence and its impact on excessive daytime sleepiness and psychological well-being in the adult Taiwanese population. Qual Life Res 2008;17:1073-1080.

3. Cunnington D, Junge MF, Fernando AT. Insomnia: prevalence, consequences and effective treatment. Med J Aust 2013;199:S36-S40.

4. Sivertsen B, Krokstad S, Øverland S, Mykletun A. The epidemiology of insomnia: associations with physical and mental health. The HUNT-2 study. J Psychosom Res 2009;67:109-116.

5. Walsh JK. Clinical and socioeconomic correlates of insomnia. J Clin Psychiatry 2004;65 Suppl 8:13-19.

6. Roane BM, Taylor DJ. Adolescent insomnia as a risk factor for early adult depression and substance abuse. Sleep 2008;31:1351-356.

7. Neckelmann D, Mykletun A, Dahl AA. Chronic insomnia as a risk factor for developing anxiety and depression. Sleep 2007;30:873-880.

8. Jaussent I, Bouyer J, Ancelin M-L, Akbaraly T, Pérès K, Ritchie K, et al. Insomnia and daytime sleepiness are risk factors for depressive symptoms in the elderly. Sleep 2011;34:1103-1110.

9. Chien KL, Chen PC, Hsu HC, Su TC, Sung FC, Chen MF, et al. Habitual sleep duration and insomnia and the risk of cardiovascular events and all-cause death: report from a community-based cohort. Sleep 2010;33:177-184.

10. Kronholm E, Laatikainen T, Peltonen M, Sippola R, Partonen T. Self-reported sleep duration, all-cause mortality, cardiovascular mortality and morbidity in Finland. Sleep

For peer review only

Med 2011;12:215-221.

11. Ozminkowski RJ, Wang S, Walsh JK. The direct and indirect costs of untreated insomnia in adults in the United States. Sleep 2007;30:263-273.
12. Vgontzas AN, Liao D, Pejovic S, Calhoun S, Karataraki M, Basta M, et al. Insomnia with short sleep duration and mortality: the Penn State cohort. Sleep 2010;33:1159-1164.
13. Gangwisch JE, Heymsfiel SB, Boden-Albala B, Buijs RM, Kreier F, Pickering TG, et al. Sleep duration as a risk factor for diabetes incidence in a large US sample. Sleep 2007;30:1667-1673.
14. Gangwisch JE, Malaspina D, Boden-Albala B, Heymsfiel SB. Inadequate sleep as a risk factor for obesity: analyses of the NHANES I. Sleep 2005;28:1289-1296.
15. Smolensky MH, Milia LD, Ohayon MM, Philip P. Sleep disorders, medical conditions, and road accident risk. Accid Anal Prev 2011;43:533-548.
16. Global Initiative for Asthma. 2015 GINA Report, Global Strategy for Asthma Management and Prevention: p. 2. Available from: http://ginasthma.org/gina-report-global-strategy-for-asthma-management-and-prevention/
17. Meltzer LJ, Ullrich M, Szefler SJ. Sleep duration, sleep hygiene, and insomnia in adolescents with asthma. J Allergy Clin Immunol Pract 2014;2:562-569.
18. Sundbom F, Lindberg E, Bjerg A, Forsberg B, Franklin K, Gunnbjörnsdottir M, et al. Asthma symptoms and nasal congestion as independent risk factors for insomnia in a general population: results from the GA(2)LEN survey. Allergy 2013;68:213-219.
19. Budhiraja R, Roth T, Hudgel DW, Budhiraja P, Drake CL. Prevalence and polysomnographic correlates of insomnia comorbid with medical disorders. Sleep 2011;34:859-867.
20. Sivertsen B, Lallukka T, Salo P, Pallesen S, Hysing M, Krokstad S, et al. Insomnia as a

For peer review only

risk factor for ill health: results from the large population-based prospective HUNT Study in Norway. J Sleep Res 2014;23:124-132.

21. Wu TJ, Wu CF, Lee YL, Hsiue TR, Guo YL. Asthma incidence, remission, relapse and persistence: a population-based study in southern Taiwan. Respir Res 201;15:135.

22. Zhang J, Lama SP, Li SX, Yu MWM, Li AM, Ma RCW, et al. Long-term outcomes and predictors of chronic insomnia: a prospective study in Hong Kong Chinese adults. Sleep Med 2012;13:455-462.

23. Burgos I, Richter L, Klein T, Fiebich B, Feige B, Lieb K, et al. Increased nocturnal interleukin-6 excretion in patients with primary insomnia: a pilot study. Brain Behav Immun 2006;20:246-253.

24. Voderholzer U, Fiebich BL, Dersch R, Feige B, Piosczyk H, Kopasz M, et al. Effects of sleep deprivation on nocturnal cytokine concentrations in depressed patients and healthy control subjects. J Neuropsychiatry Clin Neurosci 2012;24:354-366.

25. Neveu WA, Allard JL, Raymond DM, Bourassa LM, Burns SM, Bunn JY, et al. Elevation of IL-6 in the allergic asthmatic airway is independent of inflammation but associates with loss of central airway function. Respir Res 2010;11:28.

26. Yokoyama A, Kohno N, Fujino S, Hamada H, Inoue Y, Fujioka S, et al. Circulating interleukin-6 levels in patients with bronchial asthma. Am J Respir Crlt Care Med 1995;151:1354-1358.

27. Rincon M, Irvin CG. Role of IL-6 in asthma and other inflammatory pulmonary diseases. Int J Biol Sci 2012;8:1281-1290.

28. Neveu WA, Allard JB, Dienz O, Wargo MJ, Ciliberto G, Whittaker LA, et al. IL-6 is required for airway mucus production induced by inhaled fungal allergens. J Immunol 2009;183:1732-1738.

29. Irwin MR, Wang M, Ribeiro D, Cho HJ, Olmstead R, Breen EC, et al. Sleep loss

For peer review only

activates cellular inflammatory signaling. Biol Psychiatry 2008;64:538-540.

30. Ather JL, Hodgkins SR, Janssen-Heininger YMW, Poynter ME. Airway epithelial NF-kappa B activation promotes allergic sensitization to an innocuous inhaled antigen. Am J Respir Cell Mol Biol 2011;44:631-638.

31. Sakami S, Ishikawa T, Kawakami N, Haratani T, Fukui A, Kobayashi F, et al. Coemergence of insomnia and a shift in the Th1/Th2 balance toward Th2 dominance. Neuroimmunomodulation 2002-2003;10:337-343.

32. Lama M, Chatterjee M, Nayakc CR, Chaudhuri TK. Increased interleukin-4 and decreased interferon-gamma levels in serum of children with asthma. Cytokine 2011;55:335-338.

33. Mitchell C, Provost K, Niu N, Homer R, Cohn L. IFN-gamma acts on the airway epithelium to inhibit local and systemic pathology in allergic airway disease. J Immunol 2011;187:3815-3820.

For peer review only

STROBE 2007 (v4) Statement—Checklist of items that should be included in reports of *cohort studies*

Section/Topic	Item #	Recommendation	Reported on page #
**Title and abstract**	1	(*a*) Indicate the study's design with a commonly used term in the title or the abstract	1,2
		(*b*) Provide in the abstract an informative and balanced summary of what was done and what was found	2
**Introduction**			
Background/rationale	2	Explain the scientific background and rationale for the investigation being reported	4
Objectives	3	State specific objectives, including any prespecifid hypotheses	4
**Methods**			
Study design	4	Present key elements of study design early in the paper	4-6
Setting	5	Describe the setting, locations, and relevant dates, including periods of recruitment, exposure, follow-up, and data collection	4-6
Participants	6	(*a*) Give the eligibility criteria, and the sources and methods of selection of participants. Describe methods of follow-up	4-6
		(*b*) For matched studies, give matching criteria and number of exposed and unexposed	4-6
Variables	7	Clearly define all outcomes, exposures, predictors, potential confounders, and effect modifiers. Give diagnostic criteria, if applicable	4-6
Data sources/ measurement	8*	For each variable of interest, give sources of data and details of methods of assessment (measurement). Describe comparability of assessment methods if there is more than one group	4-6
Bias	9	Describe any efforts to address potential sources of bias	4-6
Study size	10	Explain how the study size was arrived at	4-6
Quantitative variables	11	Explain how quantitative variables were handled in the analyses. If applicable, describe which groupings were chosen and why	4-6
Statistical methods	12	(*a*) Describe all statistical methods, including those used to control for confounding	4-6
		(*b*) Describe any methods used to examine subgroups and interactions	4-6
		(*c*) Explain how missing data were addressed	4-6
		(*d*) If applicable, explain how loss to follow-up was addressed	4-6
		(*e*) Describe any sensitivity analyses	4-6
**Results**			

For peer review only

Participants	13*	(a) Report numbers of individuals at each stage of study—eg numbers potentially eligible, examined for eligibility, confirmed eligible, included in the study, completing follow-up, and analysed	6-12
		(b) Give reasons for non-participation at each stage	6-12
		(c) Consider use of a flow diagram	6-12
Descriptive data	14*	(a) Give characteristics of study participants (eg demographic, clinical, social) and information on exposures and potential confounders	6-12
		(b) Indicate number of participants with missing data for each variable of interest	6-12
		(c) Summarise follow-up time (eg, average and total amount)	6-12
Outcome data	15*	Report numbers of outcome events or summary measures over time	6-12
Main results	16	(*a*) Give unadjusted estimates and, if applicable, confounder-adjusted estimates and their precision (eg, 95% confidence interval). Make clear which confounders were adjusted for and why they were included	6-12
		(*b*) Report category boundaries when continuous variables were categorized	6-12
		(*c*) If relevant, consider translating estimates of relative risk into absolute risk for a meaningful time period	6-12
Other analyses	17	Report other analyses done—eg analyses of subgroups and interactions, and sensitivity analyses	6-12
**Discussion**			
Key results	18	Summarise key results with reference to study objectives	12
**Limitations**			
Interpretation	20	Give a cautious overall interpretation of results considering objectives, limitations, multiplicity of analyses, results from similar studies, and other relevant evidence	13-14
Generalisability	21	Discuss the generalisability (external validity) of the study results	14
**Other information**			
Funding	22	Give the source of funding and the role of the funders for the present study and, if applicable, for the original study on which the present article is based	15

*Give information separately for cases and controls in case-control studies and, if applicable, for exposed and unexposed groups in cohort and cross-sectional studies.

Note: An Explanation and Elaboration article discusses each checklist item and gives methodological background and published examples of transparent reporting. The STROBE checklist is best used in conjunction with this article (freely available on the Web sites of PLoS Medicine at http://www.plosmedicine.org/, Annals of Internal Medicine at http://www.annals.org/, and Epidemiology at http://www.epidem.com/). Information on the STROBE Initiative is available at www.strobe-statement.org.

For peer review only

BMJ Open

Is Insomnia a Risk Factor for New-Onset Asthma? A Population-Based Study

Journal:	*BMJ Open*
Manuscript ID	bmjopen-2017-018714.R1
Article Type:	Research
Date Submitted by the Author:	17-Oct-2017
Complete List of Authors:	Lin, Yu-Chieh; Jiannren Hospital, Family Medicine<br>Lai, Chih-Cheng; Chi Mei Medical Center, Liouying, Intensive Care Medicine<br>Chien, Chih-Chiang ; Chi Mei Medical Center, Internal Medicine<br>Chen, Chin-ming; Intensive Care Medicine<br>Chiang, Shyh-Ren; Chi Mei Medical Center, Internal Medicine<br>Ho, Chung-Han; Chi Mei Medical Center, Medical Research<br>Weng, Shih-Feng; Chi Mei Medical Center, Departmetn of Medical Research<br>Cheng, Kuo-Chen; Chi Mei Medical Center, Internal Medicine
**Primary Subject Heading**:	Epidemiology
Secondary Subject Heading:	Respiratory medicine
Keywords:	EPIDEMIOLOGY, Asthma < THORACIC MEDICINE, SLEEP MEDICINE

SCHOLARONE™ Manuscripts

For peer review only

Research Paper

Is Insomnia a Risk Factor for New-Onset Asthma? A Population-Based Study

Running title: Insomnia and Risk of Asthma

Yu-Chieh Lin, MD[1]; Chih-Cheng Lai, MD[2]*; Chih-Chiang Chien, MD[3]; Chin-Ming Chen, MD[4,5]; Shyh-Ren Chiang, MD[3,5]; Chung-Han Ho, PhD[6,7]; Shih-Feng Weng, PhD[8]; Kuo-Chen Cheng, MD[3,9]*

[1]Department of Family Medicine, Jiannren Hospital, Kaohsiung, Taiwan; [2]Department of Intensive Care Medicine, Chi Mei Medical Center, Liouying, Taiwan; Departments of [3]Internal Medicine, [4]Intensive Care Medicine, and [6]Medical Research, Chi Mei Medical Center, Tainan, Taiwan; Departments of [5]Recreation and Healthcare Management, and [7]Pharmacy, Chia Nan University of Pharmacy & Science, Tainan, Taiwan; [8]Department of Healthcare Administration and Medical Informatics, Kaohsiung Medical University, Kaohsiung, Taiwan; and [9]Department of Safety, Health, and Environmental Engineering, Chung Hwa University of Medical Technology, Tainan, Taiwan

*The first and second authors contributed equally to this work.

Correspondence: Kuo-Chen Cheng, MD, Department of Internal Medicine, Chi Mei Medical Center, Tainan, Taiwan. 901 Zhonghua Road, Yong Kang District, Tainan City 710, Taiwan

Tel: 886-6-281-2811 Ext 57111; Fax: 886-6-282-8928; E-mail: kcg.cheng@gmail.com

Financial disclosures

This research received no specific grant from any funding agency in the public, commercial or not-for-profit sectors.

Conflicts of interest

None

For peer review only

Abstract

Objectives: To determine whether insomnia at baseline is a risk factor for new-onset asthma.

Methods: We recruited 48,871 patients with insomnia (Insomnia group) newly diagnosed between 2002 and 2007, and 97,742 matched controls without insomnia (Control group) from Taiwan's Longitudinal Health Insurance Database 2000. All of the patients were followed up for 4 years to see whether new-onset asthma developed. Patients with previous asthma or insomnia were excluded. The Poisson regression was used to estimate the incidence rate ratios (IRRs) and 95% confidence intervals (CIs) of asthma. Cox proportional hazard regression was used to calculate the risk of asthma between the two groups.

Results: After a 4-year follow-up, 424 patients in the Insomnia group and 409 in the Control group developed asthma. The incidence rate of asthma was significantly higher in the insomnia group (22.01 vs. 10.57 per 10,000 person-years). Patients with insomnia have a higher risk of developing new-onset asthma during the 4-year follow-up (hazard ratio [HR]: 2.08, 95% CI: 1.82-2.39). The difference remained significant after adjustment (adjusted HR: 1.89, 95% CI: 1.64-2.17).

Conclusions: This large population-based study suggests that insomnia at baseline is a risk factor for developing asthma.

Keywords: Insomnia, asthma, comorbid, medical disorders, risk.

For peer review only

Strengths and Limitations of this study

1. The NHIRD database does not include some possible confounding factors, such as smoking habits, house dust mite exposure, etc..
2. The NHIRD database NHIRD does not contain patient information before 1996, some of our patients might have been misclassified if they were diagnosed with asthma or insomnia before that year.

For peer review only

Introduction

Insomnia is a sleep disorder that makes falling asleep or staying asleep difficult, or prevents people who awaken early in the morning from returning to sleep at least three nights per week for at least three months, despite an adequate opportunity for sleep.[1] The prevalence of insomnia is about 25% in Taiwanese adults[2]; and other countries report prevalences ranging from 9% to 50%.[3-5] Insomnia has long been linked with multiple physical conditions, allergies, cancer, hypertension, diabetes, migraine, headache, osteoporosis, fibromyalgia, rheumatoid arthritis, arthrosis, musculoskeletal disorders, and obesity.[4] Moreover, impaired sleep is related to developing anxiety and depression.[6-8] It also burdens the public health sector, reduces quality of life, and leads to more traffic accidents.[9-15]

Asthma is a heterogeneous disease characterized by recurrent attacks of breathlessness and wheezing caused by chronic airway inflammation.[16] Patients with severe asthma may have the problems of insufficient sleep, poor sleep hygiene, and clinically significant insomnia.[17,18] Although it is well known that insomnia is more prevalent in patients with asthma,[4,19] Sivertsen et al.[20] reported that the association might be bidirectional. They did not, however, focus on a single disease, and whether a respondent had asthma was based only on answers from a personal questionnaire rather than on a professional diagnosis. Nor did the study adjust for confounding factors. Therefore, we did a prospective cohort study based on clinical diagnoses to determine whether insomnia is a risk factor for new-onset asthma.

Materials and Methods

Study design and cohorts

Taiwan launched a single-payer National Health Insurance (NHI) program on March 1, 1995. The NHI databases, one of the largest and most complete population-based datasets in

For peer review only

the world, include medical claims information on almost the entire population in Taiwan (> 98% in 2009). The data used in this study were taken from the Longitudinal Health Insurance Database 2000 (LHID2000), which contains all claims data from 1996 to 2011 of 1 million (ca. 5% of all enrollees) representative beneficiaries randomly selected from Taiwan's National Health Insurance Research Database (NHIRD) in 2000. The LHID2000 provides encrypted patient identification numbers, sex, date of birth, dates of admission and discharge, the International Classification of Diseases-9th Revision-Clinical Modification (ICD-9-CM) codes of diagnoses and procedures, details of prescriptions, and expenditure amounts for all outpatient and inpatient medical benefit claims. The institutional review board of Chi Mei Medical Center approved the study and waived the requirement of informed consent because the datasets analyzed contained only deidentified patient information.

Participants

We did a prospective cohort study with two study groups: the Insomnia group (patients with newly diagnosed insomnia from 2002 to 2007) and an age-, sex-, and index-date-matched Control group (patients without insomnia). Patients in the Insomnia group (ICD-9-CM codes: 307.41, 307.42, 780.50, 780.52) were diagnosed with insomnia during at least one inpatient hospitalization or more than 3 times during outpatient clinic visits within one year as previous studies.[21-24] Patients diagnosed with asthma (ICD-9-CM code: 493) before insomnia were excluded.

The flowchart of study subjects selection presented in Fig1. Each patient with insomnia was age-, sex-, and index-date-matched to two patients without insomnia. The index dates for the Insomnia group patients were the dates of their first registration. Those index dates were used to create index dates for each Control group patient. To investigate the risks of developing asthma during the follow-up period, we tracked each participant no more than 4 years from their index date until new-onset asthma, death, or the end of the 4-year follow-up.

For peer review only

The asthma was also defended as patients with asthma diagnosis at least one inpatient hospitalization or more than 3 times during outpatient clinic visits within one year.

Definition of comorbidities

The diagnoses of different physical and mental conditions were based on ICD-9-CM codes during at least one inpatient hospitalization or more than 3 times during outpatient clinic visits within one year before index date. Comorbidities were as follow: hypertension (401-405, 362.11, 437.2), depression (311, 296.2, 296.3, 300.4), anxiety (293.84, 300.0x, 300.10, 300.2x, 300.5, 309.21), allergic rhinitis (477.x), urticaria (708.x), atopic dermatitis (691.x), bronchiolitis (466.11, 466.19, 079.6), sleep apnea (327.23, 780.51, 780.53, 780.57), cardiovascular disease (stroke and coronary heart disease) (430-438, 410-414).

Statistical analysis

Pearson's χ^2 test was used to compare differences in the baseline characteristics and comorbid medical disorders between the Insomnia and Control cohorts. The incidence rate was calculated as the number of asthma cases during the follow-up, divided by the total person-years for each group. Poisson regression was used to estimate the incidence rate ratios (IRRs) and 95% confidence intervals (CIs) of asthma between the two groups. A Kaplan-Meier analysis of the cumulative incidence rates of asthma was plotted to describe the proportion of patients with asthma, and the log-rank test was used to analyze the differences between the two cohorts. In addition, Cox proportional hazard regression was used to calculate the risk of asthma between patients with and without insomnia during the follow-up period. SAS 9.4 (SAS Institute, Inc., Cary, NC, USA) was used for all statistical analyses. Significance was set at $P < 0.05$ (two-sided). The detectable hazard ratio of 1.02 between Insomnia group and compared controls was estimated at 90% statistical power and the probability of type I error at 0.05.

For peer review only - http://bmjopen.bmj.com/site/about/guidelines.xhtml

Results

The Insomnia group contained 48,871 patients and the Control group contained 97,742 (Table 1). Insomnia was more common in women (59.96%) and in the middle-aged group (35-49 years: 33.29%). Insomnia group patients were more likely to have comorbidities, such as hypertension (HTN) (21.72% vs. 15.12%), anxiety/depression (10.15% vs. 1.69%), allergic rhinitis (3.56% vs. 1.60%), urticaria (2.37% vs. 0.97%), atopic dermatitis (0.51% vs. 0.31%), sleep apnea (0.18% vs. 0.02%), and cardiovascular disease (CVD) (10.10% vs. 6.33%) than were Control group patients. The Insomnia group tended to have lower incomes than did the Control group.

Table 1. Baseline demographic characteristics of patients in the Insomnia and Control groups

	Insomnia (n = 48,871) n (%)	Control (n = 97,742) n (%)	$P^{†}$
**Age Group (years)**			
< 34	9515 (19.47)	19,031 (19.47)	1.00
35-49	16,269 (33.29)	32,541 (33.29)	
50-64	13,611 (27.85)	27,221 (27.85)	
≥ 65	9476 (19.39)	18,949 (19.39)	
**Sex**			
Male	19,569 (40.04)	39,138 (40.04)	1.00
Female	29,302 (59.96)	58,604 (59.96)	
**Comorbidity**			
Hypertension	10,616 (21.72)	14,781 (15.12)	< 0.001
Anxiety/Depression	4961 (10.15)	1649 (1.69)	< 0.001
Allergic rhinitis	1742 (3.56)	1561 (1.60)	< 0.001
Urticaria	1158 (2.37)	949 (0.97)	< 0.001
Atopic dermatitis	247 (0.51)	299 (0.31)	< 0.001
Bronchiolitis	57 (0.12)	130 (0.13)	0.44
Sleep apnea	87 (0.18)	21 (0.02)	< 0.001
Cardiovascular disease	4936 (10.10)	6187 (6.33)	< 0.001
**Geographic Region**			
North	22,674 (46.40)	46,019 (47.08)	< 0.001
Central	11,144 (22.80)	17,352 (17.75)	
South	13,803 (28.24)	32,264 (33.01)	
East	1250 (2.56)	2107 (2.16)	

For peer review only

**SES (monthly insurable wage in NT$)***			
< 20,000	36,415 (74.51)	69,456 (71.06)	< 0.001
20,000-40,000	8370 (17.13)	18,507 (18.93)	
> 40,000	4086 (8.36)	9779 (10.00)	

SES = socioeconomic status; NT$ = New Taiwan dollar.
* US$1 NT$30.
† *P* determined using χ^2 tests

After a 4-year follow-up, 424 patients in the Insomnia group and 409 patients in the Control group had developed asthma. The incidence rate of asthma was significantly higher in the Insomnia group (22.01 vs. 10.57 per 10,000 person-years; IRR: 2.08 (95% CI: 1.82-2.39; P < 0.001) (Table 2). Patients with insomnia had a higher probability of developing asthma during the 4-year follow-up (HR: 2.08; 95% CI: 1.82-2.39). This difference was still significant after adjustment (adjusted (A)HR: 1.89; 95% CI: 1.64-2.17) (Table 3). Advanced age was also related to a higher risk for developing asthma (≥ 65 years: AHR: 8.13; 95% CI: 5.89-11.23). Patients with higher incomes were less likely to develop asthma (New Taiwan dollars (NT$) > 40,000: AHR = 0.62; 95% CI: 0.44-0.89). People living in eastern Taiwan were more likely to have new-onset asthma then those living in other regions of Taiwan (AHR = 2.01; 95% CI: 1.44-2.81). The subgroup analysis showed that insomnia was associated with a greater risk of asthma in patients with HTN and CVD (Table 4). A Kaplan-Meier survival curve shows that the Insomnia group had a higher cumulative incidence rate of asthma than did the Control group (P < 0.001) (Figure 2).

For peer review only

Table 2. Risks of asthma in the Insomnia and Control groups

Characteristics	Insomnia				Control				IRR (95% CI)	$P^{\dagger}$
	No.	Asthma	PY	IR*	No.	Asthma	PY	IR*		
**All**	48871	424	192623.41	22.01	97742	409	387095.85	10.57	2.08 (1.82-2.39)	< 0.001
**Age Group (years)**										
< 34	9515	27	37931.78	7.12	19031	17	76049.26	2.24	3.18 (1.74-5.84)	< 0.001
35-49	16269	82	64569.83	12.70	32541	32	129858.42	2.46	5.15 (3.43-7.75)	< 0.001
50-64	13611	85	53788.73	15.80	27221	83	108051.32	7.68	2.06 (1.52-2.78)	< 0.001
≥ 65	9476	230	36333.07	63.30	18949	277	73136.86	37.87	1.67 (1.40-1.99)	< 0.001
**Sex**										
Male	19569	180	76611.01	23.50	39138	179	154370.93	11.60	2.03 (1.65-2.49)	< 0.001
Female	29302	244	116012.41	21.03	58604	230	232724.93	9.88	2.13 (1.78-2.55)	< 0.001
**Comorbidity**										
Hypertension	10616	180	41264.43	43.62	14781	167	57535.99	29.03	1.50 (1.22-1.86)	< 0.001
Anxiety/Depression	4961	59	19475.68	30.29	1649	15	6417.10	23.38	1.30 (0.74-2.28)	0.37
Allergic rhinitis	1742	19	6881.46	27.61	1561	8	6157.11	12.99	2.13 (0.93-4.85)	0.07
Urticaria	949	6	3719.76	16.13	1158	6	4556.88	13.17	1.23 (0.40-3.80)	0.73
Atopic dermatitis	247	1	973.35	10.27	299	4	1152.70	34.70	0.30 (0.03-2.65)	0.28
Bronchiolitis	57	1	227.06	44.04	130	1	515.46	19.40	2.27 (0.14-6.29)	0.56
Sleep apnea	87	1	344.54	29.02	21	1	80.21	124.70	0.23 (0.01-3.72)	0.30
CVD	4936	117	18838.57	62.11	6187	114	23618.24	48.27	1.29 (0.99-1.67)	0.06

PY = person-years; IR = incidence rate; IRR = incidence rate ratio; CI = confidence interval; CVD = cardiovascular disease.

* IR: per 10,000 person-years.

† P-values were determined using Poisson regression models.

Table 3. Cox proportional hazard regressions for the development of asthma during the follow-up period

Variable	Crude HR (95% CI)	Adjusted HR (95% CI)†
**Insomnia**		
No	Ref	Ref
Yes	2.08 (1.82-2.39)	1.89 (1.64-2.17)
**Age Group**		
< 34	Ref	Ref
35-49	1.52 (1.07-2.15)	1.57 (1.11-2.22)
50-64	2.69 (1.93-3.75)	2.45 (1.75-3.43)
≥ 65	11.95 (8.78-16.26)	8.13 (5.89-11.23)
**Sex**		
Male	Ref	Ref
Female	0.88 (0.76-1.00)	1.00 (0.87-1.15)
**Comorbidity**		
Hypertension	3.47 (3.02-3.98)	1.24 (1.06-1.45)
Anxiety/Depression	2.08 (1.64-2.65)	1.19 (0.93-1.53)
Allergic rhinitis	1.46 (0.99-2.14)	1.33 (0.90-1.96)
Urticaria	1.01 (0.57-1.78)	0.81 (0.46-1.44)
Atopic dermatitis	1.64 (0.68-3.95)	1.15 (0.48-2.78)
Bronchiolitis	1.88 (0.47-7.52)	2.09 (0.52-8.38)
Sleep apnea	3.29 (0.82-13.17)	2.82 (0.70-11.37)
Cardiovascular disease	4.84 (4.16-5.63)	1.80 (1.52-2.14)
**Geographic Region**		
North	Ref	Ref
Central	1.29 (1.08-1.55)	1.26 (1.05-1.51)
South	1.28 (1.09-1.50)	1.18 (1.00-1.38)
East	2.45 (1.68-3.29)	2.01 (1.44-2.81)
**SES (monthly insurable wage in NT$)***		
< 20,000	Ref	Ref
20,000-40,000	0.27 (0.21-0.36)	0.53 (0.40-0.71)
> 40,000	0.35 (0.25-0.49)	0.62 (0.44-0.89)

HR = hazard ratio; CI = confidence interval; Ref = reference value; SES = socioeconomic status.

† Parameters were adjusted for all covariates included in the model.

* US$1 NT$30.

For peer review only

Table 4. Stratified analysis for patients with hypertension or cardiovascular disease

	Patients with HTN (n = 25397) AHR (95% CI)	*P*	Patients with CVD (n = 11123) AHR (95% CI)	*P*
**Control**	1.00 (ref.)		1.00 (ref.)	
**Insomnia**	1.59 (1.28-1.97)	< 0.001	1.38 (1.06-1.79)	0.02

HTN = hypertension; CVD = cardiovascular disease; AHR = adjusted hazard ratio; CI = confidence interval; ref. = reference value.

For peer review only

Discussion

This is the largest cohort study focused on the association between insomnia and new-onset asthma, and the first one on an Asian population. We found that insomnia patients who sought medical assistance had a significant risk for developing asthma within 4 years of asking for treatment. In this study, the incidence of asthma in control group was 10.57 per 10,000 person-years. This finding is similar to the most recent epidemiology study[25] in Taiwan that the incidence of asthma was 9.8 per 10,000 person-year. In contrast, the insomnia group had significant higher incidence of asthma – 22.01 per 10,000 person-year than the control group and the general population in previous study.[25] Several studies have reported linkages between insomnia and chronic illnesses, including asthma. A cross-sectional study[19] of 3283 adults showed a higher prevalence of insomnia in those with asthma (AOR 1.6; 95% CI: 1.3-2.0). A 5-year prospective study[26] of 2316 middle-aged adults reported that patients with insomnia at baseline had a higher incidence of asthma (AOR = 17.9; 95% CI: 2.28-140) than did patients without insomnia. But no causal relationship between insomnia and asthma was supported in these two studies. Unlike those two studies, our study excluded patients who had previously been diagnosed with asthma, and we followed our patients for 4 years, which provided evidence that insomnia is a risk factor for developing asthma.

Sivertsen et al.[20] reported that insomnia was a significant risk factor for incidence of

asthma in an 11-year large population-based prospective cohort study consisted of 24715 participants (OR = 1.47; 95% CI: 1.16-1.86). Brumpton et al.[27] also found that insomnia symptoms were associated with increased risk of incident asthma in the same population-based which consisted of 17927 participants (three insomnia symptoms, OR = 1.70; 95% CI: 1.37–2.11). However, they used questionnaires to define insomnia and provided no information about the severity or duration of their participants' poor sleep. The diagnoses of asthma and comorbidities were based on self-reports instead of physician-reported diagnoses. Their sample size was smaller than was ours, and the confounding factors adjusted for in their analysis did not include specific asthma or insomnia-related comorbidities such as atopy. In contrast, we used ICD-9 CM codes from Taiwan's LHID2000, which indicated the patients' physician-diagnosed illnesses, including insomnia, asthma, and all comorbidities. Besides, we believed that the insomniacs in our cohort suffered from more severe sleep-related symptoms that needed medical assistance. Our findings were statistically significant after our analyses had been fully adjusted for the most common confounding factors related to asthma and insomnia.

Although our findings and those of other studies[17,18,20] indicate a significant association between insomnia and asthma, the pathophysiology of insomnia-related asthma is still unclear. Nonetheless, the common inflammatory pathway between insomnia and asthma should be considered, and several mechanisms have been proposed to explain the potential relationship between insomnia and asthma. First, poor sleep increases Interleukin-6 (IL-6) production, and this response lasts until daytime.[28,29] Patients with stable and with acute asthma had significantly higher IL-6 production levels in serum, sputum, and bronchoalveolar lavage fluid than did healthy controls.[30,31] IL-6 production in the airway promotes allergic airway inflammation in mice. In contrast, using IL-6 knockout mice in the same model showed significantly less mucus secretion.[32,33] Therefore, insomnia might contribute to inducing IL-6

For peer review only

production and that might exacerbate airway hypersensitivity. Second, NF-κB can be induced by sleep loss.[34] Prolonged airway epithelial NF-κB activation has been reported in patients with asthma. Even temporal NF-κB activation in the airway epithelium is sufficient to induce airway hyperresponsiveness in mice.[35] Thus, the higher incidence of asthma in insomnia patients might be the result of temporal or persistent NF-κB activation induced by sleep loss. Third, insomnia is related to a reduction in interferon (IFN)-γ.[36] IFN-γ production, which inhibits airway epithelial inflammation, is lower in asthma patients than in healthy controls.[37,38] These studies suggest that IFN-γ plays a significant role between insomnia and asthma.

The strengths of our study are its prospective cohort design and that we used the LHID2000, a subset of Taiwan's National Health Insurance Research Database (NHIRD), which contains physician-provided clinical diagnoses instead of illnesses self-reported by patients. The LHID2000 reflects the real-world situation in Taiwan and is more generalizable than are hospital-based or city-based databases. The large sample size and long-term follow-up also provide considerable statistical power.

Our study also has some limitations. First, the NHIRD does not include results of pulmonary function tests or blood tests for inflammatory cytokines, severity levels or the actual duration of insomnia, some important confounding factors, such as exposure to house dust mites, smoking habits, body mass index, family history, or medical compliance. Second, we did not assess the association between the medication for insomnia and asthma. However, it would be interesting to note if there were any differences in the development of asthma in those insomnia patients who were treated versus those who were not. Further study is warranted to investigate the drug effect. Third, some patients might have minor asthmatic symptoms and have not been diagnosed yet before insomnia. Forth, sampling bias, such as a higher number of individuals likely to seek medical care among insomnia group than control

For peer review only

group is possible and it may confound the analysis. Finally, because the NHIRD does not contain patient information before 1996, some of our patients might have been misclassified if they were diagnosed with asthma or insomnia before that year.

Conclusion

We found that insomnia patients who required medical assistance had a higher risk for developing new-onset asthma. Proper treatments for insomnia patients might help prevent the progress of airway inflammation. Additional study is needed to identify the actual mechanism that connects insomnia and asthma.

Author contributions:

YCL, CCL, and KCC designed the study, interpreted the data and drafted, and revised the article. CCC, CMC, and SRC contributed to interpreting the data and revising the article. CHH and SFW contributed to the statistical analysis. KCC critically reviewed and revised the article. All of the authors read and agreed with the final version of the manuscript.

Data sharing statement:

The data on the study population that were obtained from the NHIRD (https://nhird.nhri.org.tw/en/) are maintained in the NHRI (http://nhird.nhri.org.tw/). The NHIRD is limited for research purposes only. Applicants must follow the Computer-Processed Personal Data Protection Law (http://www.winklerpartners.com/?p=987) and related regulations of National Health Insurance Administration. All applications are reviewed for approval of data release. Interested researchers may submit queries related to data access to nhird@nhri.org.tw

For peer review only

Figure Legends

Figure 1.The flowchart of study subjects selection

Figure 2. The probability of developing asthma in insomnia and control group

For peer review only

References

1. American Psychiatric Association. Diagnostic and statistical manual of mental disorders, fifth edition. Arlington, VA: Author, 2013.
2. Kao CC, Huang CJ, Wang MY, Tsai PS. Insomnia: prevalence and its impact on excessive daytime sleepiness and psychological well-being in the adult Taiwanese population. Qual Life Res 2008;17:1073-1080.
3. Cunnington D, Junge MF, Fernando AT. Insomnia: prevalence, consequences and effective treatment. Med J Aust 2013;199:S36-S40.
4. Sivertsen B, Krokstad S, Øverland S, Mykletun A. The epidemiology of insomnia: associations with physical and mental health. The HUNT-2 study. J Psychosom Res 2009;67:109-116.
5. Walsh JK. Clinical and socioeconomic correlates of insomnia. J Clin Psychiatry 2004;65 Suppl 8:13-19.
6. Roane BM, Taylor DJ. Adolescent insomnia as a risk factor for early adult depression and substance abuse. Sleep 2008;31:1351-356.
7. Neckelmann D, Mykletun A, Dahl AA. Chronic insomnia as a risk factor for developing anxiety and depression. Sleep 2007;30:873-880.
8. Jaussent I, Bouyer J, Ancelin M-L, Akbaraly T, Pérès K, Ritchie K, et al. Insomnia and daytime sleepiness are risk factors for depressive symptoms in the elderly. Sleep 2011;34:1103-1110.
9. Chien KL, Chen PC, Hsu HC, Su TC, Sung FC, Chen MF, et al. Habitual sleep duration and insomnia and the risk of cardiovascular events and all-cause death: report from a community-based cohort. Sleep 2010;33:177-184.
10. Kronholm E, Laatikainen T, Peltonen M, Sippola R, Partonen T. Self-reported sleep duration, all-cause mortality, cardiovascular mortality and morbidity in Finland. Sleep

For peer review only

Med 2011;12:215-221.

11. Ozminkowski RJ, Wang S, Walsh JK. The direct and indirect costs of untreated insomnia in adults in the United States. Sleep 2007;30:263-273.

12. Vgontzas AN, Liao D, Pejovic S, Calhoun S, Karataraki M, Basta M, et al. Insomnia with short sleep duration and mortality: the Penn State cohort. Sleep 2010;33:1159-1164.

13. Gangwisch JE, Heymsfiel SB, Boden-Albala B, Buijs RM, Kreier F, Pickering TG, et al. Sleep duration as a risk factor for diabetes incidence in a large US sample. Sleep 2007;30:1667-1673.

14. Gangwisch JE, Malaspina D, Boden-Albala B, Heymsfiel SB. Inadequate sleep as a risk factor for obesity: analyses of the NHANES I. Sleep 2005;28:1289-1296.

15. Smolensky MH, Milia LD, Ohayon MM, Philip P. Sleep disorders, medical conditions, and road accident risk. Accid Anal Prev 2011;43:533-548.

16. Global Initiative for Asthma. 2015 GINA Report, Global Strategy for Asthma Management and Prevention: p. 2. Available from: http://ginasthma.org/gina-report-global-strategy-for-asthma-management-and-prevention/

17. Meltzer LJ, Ullrich M, Szefler SJ. Sleep duration, sleep hygiene, and insomnia in adolescents with asthma. J Allergy Clin Immunol Pract 2014;2:562-569.

18. Sundbom F, Lindberg E, Bjerg A, Forsberg B, Franklin K, Gunnbjörnsdottir M, et al. Asthma symptoms and nasal congestion as independent risk factors for insomnia in a general population: results from the GA(2)LEN survey. Allergy 2013;68:213-219.

19. Budhiraja R, Roth T, Hudgel DW, Budhiraja P, Drake CL. Prevalence and polysomnographic correlates of insomnia comorbid with medical disorders. Sleep 2011;34:859-867.

20. Sivertsen B, Lallukka T, Salo P, Pallesen S, Hysing M, Krokstad S, et al. Insomnia as a

For peer review only

risk factor for ill health: results from the large population-based prospective HUNT Study in Norway. J Sleep Res 2014;23:124-132.

21. Chen PJ, Huang CLC, Weng SF, Wu MP, Ho CH, Wang JJ, et al. Relapse insomnia increases greater risk of anxiety and depression: evidence from a population-based 4-year cohort study. Sleep Med 2017;38:122-129
22. Huang CLC, Weng SF, Wang JJ, Hsu YW, Wu MP. Risks of treated insomnia, anxiety, and depression in health care-seeking physicians: a nationwide population-based study. Medicine 2015;94:e1323.
23. Chuang YC, Weng SF, Hsu YW, Huang CLC, Wu MP. Increased risks of healthcare-seeking behaviors of anxiety, depression and insomnia among patients with bladder pain syndrome/interstitial cystitis: a nationwide population-based study. Int Urol Nephrol 2015;47:275-281.
24. Wu MP, Lin HJ, Weng SF, Ho CH, Wang JJ, Hsu YW. Insomnia Subtypes and the Subsequent Risks of Stroke. Stroke 2014;45:1349-1354.
25. Wu TJ, Wu CF, Lee YL, Hsiue TR, Guo YL. Asthma incidence, remission, relapse and persistence: a population-based study in southern Taiwan. Respir Res 2014;15:135.
26. Zhang J, Lama SP, Li SX, Yu MWM, Li AM, Ma RCW, et al. Long-term outcomes and predictors of chronic insomnia: a prospective study in Hong Kong Chinese adults. Sleep Med 2012;13:455-462.
27. Brumpton B, Mai XM, Langhammer A, Laugsand LE, Janszky I, Strand LB. Prospective study of insomnia and incident asthma in adults: the HUNT study. European Respiratory Journal Feb 2017;49:1601327
28. Burgos I, Richter L, Klein T, Fiebich B, Feige B, Lieb K, et al. Increased nocturnal interleukin-6 excretion in patients with primary insomnia: a pilot study. Brain Behav Immun 2006;20:246-253.

For peer review only

29. Voderholzer U, Fiebich BL, Dersch R, Feige B, Piosczyk H, Kopasz M, et al. Effects of sleep deprivation on nocturnal cytokine concentrations in depressed patients and healthy control subjects. J Neuropsychiatry Clin Neurosci 2012;24:354-366.

30. Neveu WA, Allard JL, Raymond DM, Bourassa LM, Burns SM, Bunn JY, et al. Elevation of IL-6 in the allergic asthmatic airway is independent of inflammation but associates with loss of central airway function. Respir Res 2010;11:28.

31. Yokoyama A, Kohno N, Fujino S, Hamada H, Inoue Y, Fujioka S, et al. Circulating interleukin-6 levels in patients with bronchial asthma. Am J Respir Crlt Care Med 1995;151:1354-1358.

32. Rincon M, Irvin CG. Role of IL-6 in asthma and other inflammatory pulmonary diseases. Int J Biol Sci 2012;8:1281-1290.

33. Neveu WA, Allard JB, Dienz O, Wargo MJ, Ciliberto G, Whittaker LA, et al. IL-6 is required for airway mucus production induced by inhaled fungal allergens. J Immunol 2009;183:1732-1738.

34. Irwin MR, Wang M, Ribeiro D, Cho HJ, Olmstead R, Breen EC, et al. Sleep loss activates cellular inflammatory signaling. Biol Psychiatry 2008;64:538-540.

35. Ather JL, Hodgkins SR, Janssen-Heininger YMW, Poynter ME. Airway epithelial NF-kappa B activation promotes allergic sensitization to an innocuous inhaled antigen. Am J Respir Cell Mol Biol 2011;44:631-638.

36. Sakami S, Ishikawa T, Kawakami N, Haratani T, Fukui A, Kobayashi F, et al. Coemergence of insomnia and a shift in the Th1/Th2 balance toward Th2 dominance. Neuroimmunomodulation 2002-2003;10:337-343.

37. Lama M, Chatterjee M, Nayakc CR, Chaudhuri TK. Increased interleukin-4 and decreased interferon-gamma levels in serum of children with asthma. Cytokine 2011;55:335-338.

For peer review only

38. Mitchell C, Provost K, Niu N, Homer R, Cohn L. IFN-gamma acts on the airway epithelium to inhibit local and systemic pathology in allergic airway disease. J Immunol 2011;187:3815-3820.

For peer review only

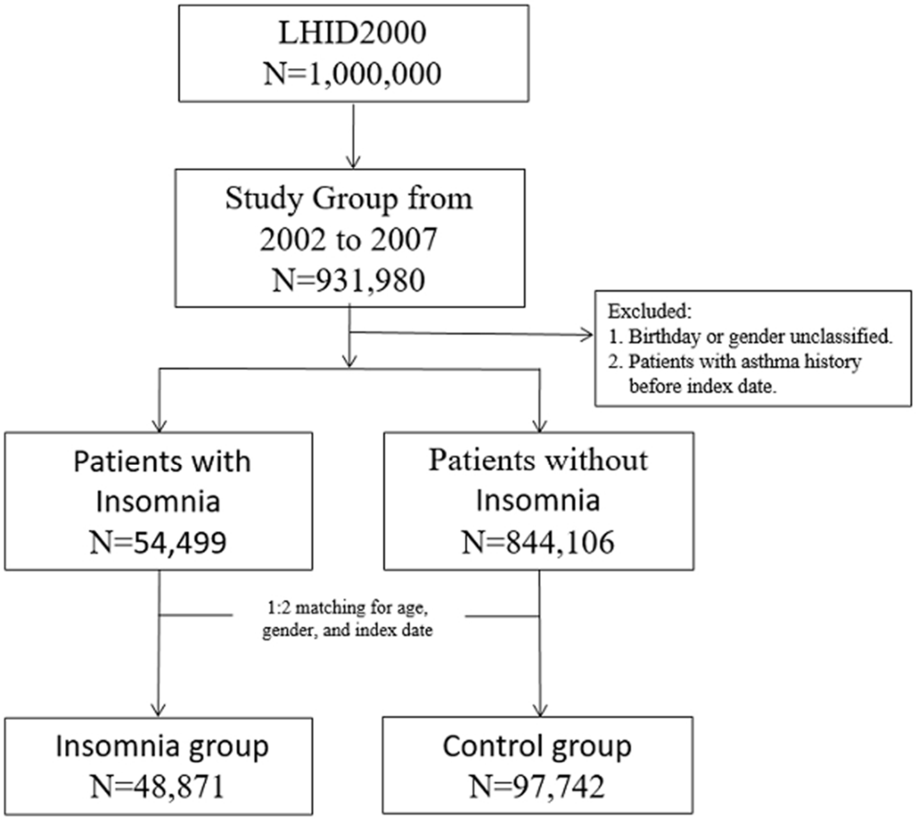

Figure 1.The flowchart of study subjects selection

86x77mm (300 x 300 DPI)

only

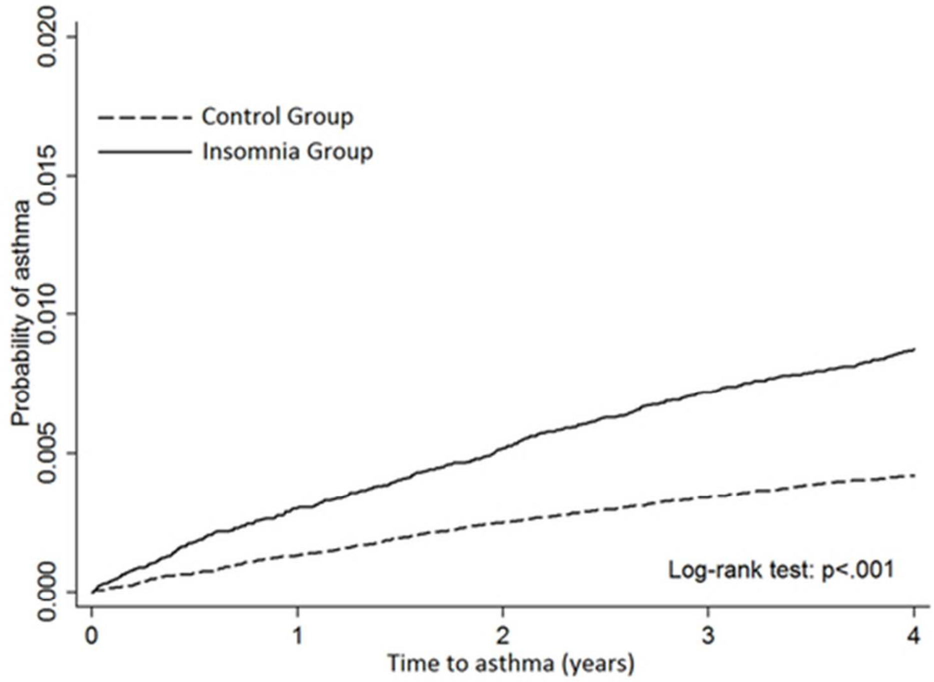

Figure 2. The probability of developing asthma in insomnia and control group

45x32mm (300 x 300 DPI)

STROBE 2007 (v4) Statement—Checklist of items that should be included in reports of *cohort studies*

Section/Topic	Item #	Recommendation	Reported on page #
**Title and abstract**	1	(*a*) Indicate the study's design with a commonly used term in the title or the abstract	1,2
		(*b*) Provide in the abstract an informative and balanced summary of what was done and what was found	2
**Introduction**			
Background/rationale	2	Explain the scientific background and rationale for the investigation being reported	4
Objectives	3	State specific objectives, including any prespecifid hypotheses	4
**Methods**			
Study design	4	Present key elements of study design early in the paper	4-6
Setting	5	Describe the setting, locations, and relevant dates, including periods of recruitment, exposure, follow-up, and data collection	4-6
Participants	6	(*a*) Give the eligibility criteria, and the sources and methods of selection of participants. Describe methods of follow-up	4-6
		(*b*) For matched studies, give matching criteria and number of exposed and unexposed	4-6
Variables	7	Clearly define all outcomes, exposures, predictors, potential confounders, and effect modifiers. Give diagnostic criteria, if applicable	4-6
Data sources/ measurement	8*	For each variable of interest, give sources of data and details of methods of assessment (measurement). Describe comparability of assessment methods if there is more than one group	4-6
Bias	9	Describe any efforts to address potential sources of bias	4-6
Study size	10	Explain how the study size was arrived at	4-6
Quantitative variables	11	Explain how quantitative variables were handled in the analyses. If applicable, describe which groupings were chosen and why	4-6
Statistical methods	12	(*a*) Describe all statistical methods, including those used to control for confounding	4-6
		(*b*) Describe any methods used to examine subgroups and interactions	4-6
		(*c*) Explain how missing data were addressed	4-6
		(*d*) If applicable, explain how loss to follow-up was addressed	4-6
		(*e*) Describe any sensitivity analyses	4-6
**Results**			

Participants	13*	(a) Report numbers of individuals at each stage of study—eg numbers potentially eligible, examined for eligibility, confirmed eligible, included in the study, completing follow-up, and analysed	6-12
		(b) Give reasons for non-participation at each stage	6-12
		(c) Consider use of a flow diagram	6-12
Descriptive data	14*	(a) Give characteristics of study participants (eg demographic, clinical, social) and information on exposures and potential confounders	6-12
		(b) Indicate number of participants with missing data for each variable of interest	6-12
		(c) Summarise follow-up time (eg, average and total amount)	6-12
Outcome data	15*	Report numbers of outcome events or summary measures over time	6-12
Main results	16	(*a*) Give unadjusted estimates and, if applicable, confounder-adjusted estimates and their precision (eg, 95% confidence interval). Make clear which confounders were adjusted for and why they were included	6-12
		(*b*) Report category boundaries when continuous variables were categorized	6-12
		(*c*) If relevant, consider translating estimates of relative risk into absolute risk for a meaningful time period	6-12
Other analyses	17	Report other analyses done—eg analyses of subgroups and interactions, and sensitivity analyses	6-12
**Discussion**			
Key results	18	Summarise key results with reference to study objectives	12
**Limitations**			
Interpretation	20	Give a cautious overall interpretation of results considering objectives, limitations, multiplicity of analyses, results from similar studies, and other relevant evidence	13-14
Generalisability	21	Discuss the generalisability (external validity) of the study results	14
**Other information**			
Funding	22	Give the source of funding and the role of the funders for the present study and, if applicable, for the original study on which the present article is based	15

*Give information separately for cases and controls in case-control studies and, if applicable, for exposed and unexposed groups in cohort and cross-sectional studies.

Note: An Explanation and Elaboration article discusses each checklist item and gives methodological background and published examples of transparent reporting. The STROBE checklist is best used in conjunction with this article (freely available on the Web sites of PLoS Medicine at http://www.plosmedicine.org/, Annals of Internal Medicine at http://www.annals.org/, and Epidemiology at http://www.epidem.com/). Information on the STROBE Initiative is available at www.strobe-statement.org.

For peer review only

Is Insomnia a Risk Factor for New-Onset Asthma? A Population-Based Study in Taiwan

Journal:	*BMJ Open*
Manuscript ID	bmjopen-2017-018714.R2
Article Type:	Research
Date Submitted by the Author:	03-Nov-2017
Complete List of Authors:	Lin, Yu-Chieh; Jiannren Hospital, Family Medicine<br>Lai, Chih-Cheng; Chi Mei Medical Center, Liouying, Intensive Care Medicine<br>Chien, Chih-Chiang ; Chi Mei Medical Center, Internal Medicine<br>Chen, Chin-ming; Intensive Care Medicine<br>Chiang, Shyh-Ren; Chi Mei Medical Center, Internal Medicine<br>Ho, Chung-Han; Chi Mei Medical Center, Medical Research<br>Weng, Shih-Feng; Chi Mei Medical Center, Departmetn of Medical Research<br>Cheng, Kuo-Chen; Chi Mei Medical Center, Internal Medicine
**Primary Subject Heading**:	Epidemiology
Secondary Subject Heading:	Respiratory medicine
Keywords:	EPIDEMIOLOGY, Asthma < THORACIC MEDICINE, SLEEP MEDICINE

For peer review only

Research Paper

Is Insomnia a Risk Factor for New-Onset Asthma? A Population-Based Study in Taiwan

Running title: Insomnia and Risk of Asthma

Yu-Chieh Lin, MD[1]; Chih-Cheng Lai, MD[2]*; Chih-Chiang Chien, MD[3]; Chin-Ming Chen, MD[4,5]; Shyh-Ren Chiang, MD[3,5]; Chung-Han Ho, PhD[6,7]; Shih-Feng Weng, PhD[8]; Kuo-Chen Cheng, MD[3,9]*

[1]Department of Family Medicine, Jiannren Hospital, Kaohsiung, Taiwan; [2]Department of Intensive Care Medicine, Chi Mei Medical Center, Liouying, Taiwan; Departments of [3]Internal Medicine, [4]Intensive Care Medicine, and [6]Medical Research, Chi Mei Medical Center, Tainan, Taiwan; Departments of [5]Recreation and Healthcare Management, and [7]Hospital and Health Care Administration, Chia Nan University of Pharmacy & Science, Tainan, Taiwan; [8]Department of Healthcare Administration and Medical Informatics, Kaohsiung Medical University, Kaohsiung, Taiwan; and [9]Department of Safety, Health, and Environmental Engineering, Chung Hwa University of Medical Technology, Tainan, Taiwan

*The first and second authors contributed equally to this work.

Correspondence: Kuo-Chen Cheng, MD, Department of Internal Medicine, Chi Mei Medical Center, Tainan, Taiwan. 901 Zhonghua Road, Yong Kang District, Tainan City 710, Taiwan

Tel: 886-6-281-2811 Ext 57111; Fax: 886-6-282-8928; E-mail: kcg.cheng@gmail.com

Financial disclosures

This research received no specific grant from any funding agency in the public, commercial or not-for-profit sectors.

Conflicts of interest

None

For peer review only

Abstract

Objectives: To determine whether insomnia at baseline is a risk factor for new-onset asthma.

Methods: We recruited 48,871 patients with insomnia (Insomnia group) newly diagnosed between 2002 and 2007, and 97,742 matched controls without insomnia (Control group) from Taiwan's Longitudinal Health Insurance Database 2000. All of the patients were followed up for 4 years to see whether new-onset asthma developed. Patients with previous asthma or insomnia were excluded. The Poisson regression was used to estimate the incidence rate ratios (IRRs) and 95% confidence intervals (CIs) of asthma. Cox proportional hazard regression was used to calculate the risk of asthma between the two groups.

Results: After a 4-year follow-up, 424 patients in the Insomnia group and 409 in the Control group developed asthma. The incidence rate of asthma was significantly higher in the insomnia group (22.01 vs. 10.57 per 10,000 person-years). Patients with insomnia have a higher risk of developing new-onset asthma during the 4-year follow-up (hazard ratio [HR]: 2.08, 95% CI: 1.82-2.39). The difference remained significant after adjustment (adjusted HR: 1.89, 95% CI: 1.64-2.17).

Conclusions: This large population-based study suggests that insomnia at baseline is a risk factor for developing asthma.

Keywords: Insomnia, asthma, comorbid, medical disorders, risk.

For peer review only

Strengths and Limitations of this study

1. NHIRD does not contain patient information before 1996.
2. NHIRD does not include results of pulmonary function tests or blood tests for inflammatory cytokines, severity levels or the actual duration of insomnia
3. NHIRD does not include some important confounding factors, such as exposure to house dust mites, smoking habits, body mass index, family history, or medical compliance
4. Methodology strengths of this study is the large sample size and long-term follow-up which provide considerable statistical power
5. This study can reflect the real-world situation in Taiwan and is more generalizable than are hospital-based or city-based databases.

For peer review only

Introduction

Insomnia is a sleep disorder that makes falling asleep or staying asleep difficult, or prevents people who awaken early in the morning from returning to sleep at least three nights per week for at least three months, despite an adequate opportunity for sleep.[1] The prevalence of insomnia is about 25% in Taiwanese adults[2]; and other countries report prevalences ranging from 9% to 50%.[3-5] Insomnia has long been linked with multiple physical conditions, allergies, cancer, hypertension, diabetes, migraine, headache, osteoporosis, fibromyalgia, rheumatoid arthritis, arthrosis, musculoskeletal disorders, and obesity.[4] Moreover, impaired sleep is related to developing anxiety and depression.[6-8] It also burdens the public health sector, reduces quality of life, and leads to more traffic accidents.[9-15]

Asthma is a heterogeneous disease characterized by recurrent attacks of breathlessness and wheezing caused by chronic airway inflammation.[16] Patients with severe asthma may have the problems of insufficient sleep, poor sleep hygiene, and clinically significant insomnia.[17,18] Although it is well known that insomnia is more prevalent in patients with asthma,[4,19] Sivertsen et al.[20] reported that the association might be bidirectional. They did not, however, focus on a single disease, and whether a respondent had asthma was based only on answers from a personal questionnaire rather than on a professional diagnosis. Nor did the study adjust for confounding factors. Therefore, we did a prospective cohort study based on clinical diagnoses to determine whether insomnia is a risk factor for new-onset asthma.

Materials and Methods

Study design and cohorts

Taiwan launched a single-payer National Health Insurance (NHI) program on March 1, 1995. The NHI databases, one of the largest and most complete population-based datasets in

For peer review only

the world, include medical claims information on almost the entire population in Taiwan (> 98% in 2009). The data used in this study were taken from the Longitudinal Health Insurance Database 2000 (LHID2000), which contains all claims data from 1996 to 2011 of 1 million (ca. 5% of all enrollees) representative beneficiaries randomly selected from Taiwan's National Health Insurance Research Database (NHIRD) in 2000. The LHID2000 provides encrypted patient identification numbers, sex, date of birth, dates of admission and discharge, the International Classification of Diseases-9th Revision-Clinical Modification (ICD-9-CM) codes of diagnoses and procedures, details of prescriptions, and expenditure amounts for all outpatient and inpatient medical benefit claims. The institutional review board of Chi Mei Medical Center approved the study and waived the requirement of informed consent because the datasets analyzed contained only deidentified patient information.

Participants

We did a prospective cohort study with two study groups: the Insomnia group (patients with newly diagnosed insomnia from 2002 to 2007) and an age-, sex-, and index-date-matched Control group (patients without insomnia). Patients in the Insomnia group (ICD-9-CM codes: 307.41, 307.42, 780.50, 780.52) were diagnosed with insomnia during at least one inpatient hospitalization or more than 3 times during outpatient clinic visits within one year as previous studies.[21-24] Patients diagnosed with asthma (ICD-9-CM code: 493) before insomnia were excluded.

The flowchart of study subjects selection presented in Fig1. Each patient with insomnia was age-, sex-, and index-date-matched to two patients without insomnia. The index dates for the Insomnia group patients were the dates of their first registration. Those index dates were used to create index dates for each Control group patient. To investigate the risks of developing asthma during the follow-up period, we tracked each participant no more than 4 years from their index date until new-onset asthma, death, or the end of the 4-year follow-up.

For peer review only

The asthma was also defended as patients with asthma diagnosis at least one inpatient hospitalization or more than 3 times during outpatient clinic visits within one year.

Definition of comorbidities

The diagnoses of different physical and mental conditions were based on ICD-9-CM codes during at least one inpatient hospitalization or more than 3 times during outpatient clinic visits within one year before index date. Comorbidities were as follow: hypertension (401-405, 362.11, 437.2), depression (311, 296.2, 296.3, 300.4), anxiety (293.84, 300.0x, 300.10, 300.2x, 300.5, 309.21), allergic rhinitis (477.x), urticaria (708.x), atopic dermatitis (691.x), bronchiolitis (466.11, 466.19, 079.6), sleep apnea (327.23, 780.51, 780.53, 780.57), cardiovascular disease (stroke and coronary heart disease) (430-438, 410-414).

Statistical analysis

Pearson's χ^2 test was used to compare differences in the baseline characteristics and comorbid medical disorders between the Insomnia and Control cohorts. The incidence rate was calculated as the number of asthma cases during the follow-up, divided by the total person-years for each group. Poisson regression was used to estimate the incidence rate ratios (IRRs) and 95% confidence intervals (CIs) of asthma between the two groups. A Kaplan-Meier analysis of the cumulative incidence rates of asthma was plotted to describe the proportion of patients with asthma, and the log-rank test was used to analyze the differences between the two cohorts. In addition, Cox proportional hazard regression was used to calculate the risk of asthma between patients with and without insomnia during the follow-up period. SAS 9.4 (SAS Institute, Inc., Cary, NC, USA) was used for all statistical analyses. Significance was set at $P < 0.05$ (two-sided). The detectable hazard ratio of 1.02 between Insomnia group and compared controls was estimated at 90% statistical power and the probability of type I error at 0.05.

For peer review only - http://bmjopen.bmj.com/site/about/guidelines.xhtml

Results

The Insomnia group contained 48,871 patients and the Control group contained 97,742 (Table 1). Insomnia was more common in women (59.96%) and in the middle-aged group (35-49 years: 33.29%). Insomnia group patients were more likely to have comorbidities, such as hypertension (HTN) (21.72% vs. 15.12%), anxiety/depression (10.15% vs. 1.69%), allergic rhinitis (3.56% vs. 1.60%), urticaria (2.37% vs. 0.97%), atopic dermatitis (0.51% vs. 0.31%), sleep apnea (0.18% vs. 0.02%), and cardiovascular disease (CVD) (10.10% vs. 6.33%) than were Control group patients. The Insomnia group tended to have lower incomes than did the Control group.

Table 1. Baseline demographic characteristics of patients in the Insomnia and Control groups

	Insomnia (n = 48,871) n (%)	Control (n = 97,742) n (%)	*P*[†]
**Age Group (years)**			
< 34	9515 (19.47)	19,031 (19.47)	1.00
35-49	16,269 (33.29)	32,541 (33.29)	
50-64	13,611 (27.85)	27,221 (27.85)	
≥ 65	9476 (19.39)	18,949 (19.39)	
**Sex**			
Male	19,569 (40.04)	39,138 (40.04)	1.00
Female	29,302 (59.96)	58,604 (59.96)	
**Comorbidity**			
Hypertension	10,616 (21.72)	14,781 (15.12)	< 0.001
Anxiety/Depression	4961 (10.15)	1649 (1.69)	< 0.001
Allergic rhinitis	1742 (3.56)	1561 (1.60)	< 0.001
Urticaria	1158 (2.37)	949 (0.97)	< 0.001
Atopic dermatitis	247 (0.51)	299 (0.31)	< 0.001
Bronchiolitis	57 (0.12)	130 (0.13)	0.44
Sleep apnea	87 (0.18)	21 (0.02)	< 0.001
Cardiovascular disease	4936 (10.10)	6187 (6.33)	< 0.001
**Geographic Region**			
North	22,674 (46.40)	46,019 (47.08)	< 0.001
Central	11,144 (22.80)	17,352 (17.75)	
South	13,803 (28.24)	32,264 (33.01)	
East	1250 (2.56)	2107 (2.16)	

For peer review only

**SES (monthly insurable wage in NT$)**[*]			
< 20,000	36,415 (74.51)	69,456 (71.06)	< 0.001
20,000-40,000	8370 (17.13)	18,507 (18.93)	
> 40,000	4086 (8.36)	9779 (10.00)	

SES = socioeconomic status; NT$ = New Taiwan dollar.

[*] US$1 NT$30.

[†] P determined using χ^2 tests

After a 4-year follow-up, 424 patients in the Insomnia group and 409 patients in the Control group had developed asthma. The incidence rate of asthma was significantly higher in the Insomnia group (22.01 vs. 10.57 per 10,000 person-years; IRR: 2.08 (95% CI: 1.82-2.39; P < 0.001) (Table 2). Patients with insomnia had a higher probability of developing asthma during the 4-year follow-up (HR: 2.08; 95% CI: 1.82-2.39). This difference was still significant after adjustment (adjusted (A)HR: 1.89; 95% CI: 1.64-2.17) (Table 3). Advanced age was also related to a higher risk for developing asthma (≥ 65 years: AHR: 8.13; 95% CI: 5.89-11.23). Patients with higher incomes were less likely to develop asthma (New Taiwan dollars (NT$) > 40,000: AHR = 0.62; 95% CI: 0.44-0.89). People living in eastern Taiwan were more likely to have new-onset asthma then those living in other regions of Taiwan (AHR = 2.01; 95% CI: 1.44-2.81). The subgroup analysis showed that insomnia was associated with a greater risk of asthma in patients with HTN and CVD (Table 4). A Kaplan-Meier survival curve shows that the Insomnia group had a higher cumulative incidence rate of asthma than did the Control group (P < 0.001) (Figure 2).

For peer review only

Table 2. Risks of asthma in the Insomnia and Control groups

Characteristics	Insomnia				Control				IRR (95% CI)	$P^{\dagger}$
	No.	Asthma	PY	IR*	No.	Asthma	PY	IR*		
**All**	48871	424	192623.41	22.01	97742	409	387095.85	10.57	2.08 (1.82-2.39)	< 0.001
**Age Group (years)**										
< 34	9515	27	37931.78	7.12	19031	17	76049.26	2.24	3.18 (1.74-5.84)	< 0.001
35-49	16269	82	64569.83	12.70	32541	32	129858.42	2.46	5.15 (3.43-7.75)	< 0.001
50-64	13611	85	53788.73	15.80	27221	83	108051.32	7.68	2.06 (1.52-2.78)	< 0.001
≥ 65	9476	230	36333.07	63.30	18949	277	73136.86	37.87	1.67 (1.40-1.99)	< 0.001
**Sex**										
Male	19569	180	76611.01	23.50	39138	179	154370.93	11.60	2.03 (1.65-2.49)	< 0.001
Female	29302	244	116012.41	21.03	58604	230	232724.93	9.88	2.13 (1.78-2.55)	< 0.001
**Comorbidity**										
Hypertension	10616	180	41264.43	43.62	14781	167	57535.99	29.03	1.50 (1.22-1.86)	< 0.001
Anxiety/Depression	4961	59	19475.68	30.29	1649	15	6417.10	23.38	1.30 (0.74-2.28)	0.37
Allergic rhinitis	1742	19	6881.46	27.61	1561	8	6157.11	12.99	2.13 (0.93-4.85)	0.07
Urticaria	949	6	3719.76	16.13	1158	6	4556.88	13.17	1.23 (0.40-3.80)	0.73
Atopic dermatitis	247	1	973.35	10.27	299	4	1152.70	34.70	0.30 (0.03-2.65)	0.28
Bronchiolitis	57	1	227.06	44.04	130	1	515.46	19.40	2.27 (0.14-6.29)	0.56
Sleep apnea	87	1	344.54	29.02	21	1	80.21	124.70	0.23 (0.01-3.72)	0.30
CVD	4936	117	18838.57	62.11	6187	114	23618.24	48.27	1.29 (0.99-1.67)	0.06

PY = person-years; IR = incidence rate; IRR = incidence rate ratio; CI = confidence interval; CVD = cardiovascular disease.

* IR: per 10,000 person-years.

† P-values were determined using Poisson regression models.

For peer review only

Table 3. Cox proportional hazard regressions for the development of asthma during the follow-up period

Variable	Crude HR (95% CI)	Adjusted HR (95% CI)†
**Insomnia**		
No	Ref	Ref
Yes	2.08 (1.82-2.39)	1.89 (1.64-2.17)
**Age Group**		
< 34	Ref	Ref
35-49	1.52 (1.07-2.15)	1.57 (1.11-2.22)
50-64	2.69 (1.93-3.75)	2.45 (1.75-3.43)
≥ 65	11.95 (8.78-16.26)	8.13 (5.89-11.23)
**Sex**		
Male	Ref	Ref
Female	0.88 (0.76-1.00)	1.00 (0.87-1.15)
**Comorbidity**		
Hypertension	3.47 (3.02-3.98)	1.24 (1.06-1.45)
Anxiety/Depression	2.08 (1.64-2.65)	1.19 (0.93-1.53)
Allergic rhinitis	1.46 (0.99-2.14)	1.33 (0.90-1.96)
Urticaria	1.01 (0.57-1.78)	0.81 (0.46-1.44)
Atopic dermatitis	1.64 (0.68-3.95)	1.15 (0.48-2.78)
Bronchiolitis	1.88 (0.47-7.52)	2.09 (0.52-8.38)
Sleep apnea	3.29 (0.82-13.17)	2.82 (0.70-11.37)
Cardiovascular disease	4.84 (4.16-5.63)	1.80 (1.52-2.14)
**Geographic Region**		
North	Ref	Ref
Central	1.29 (1.08-1.55)	1.26 (1.05-1.51)
South	1.28 (1.09-1.50)	1.18 (1.00-1.38)
East	2.45 (1.68-3.29)	2.01 (1.44-2.81)
**SES (monthly insurable wage in NT$)***		
< 20,000	Ref	Ref
20,000-40,000	0.27 (0.21-0.36)	0.53 (0.40-0.71)
> 40,000	0.35 (0.25-0.49)	0.62 (0.44-0.89)

HR = hazard ratio; CI = confidence interval; Ref = reference value; SES = socioeconomic status.

† Parameters were adjusted for all covariates included in the model.

* US$1 NT$30.

For peer review only

Table 4. Stratified analysis for patients with hypertension or cardiovascular disease

	Patients with HTN (n = 25397) AHR (95% CI)	*P*	Patients with CVD (n = 11123) AHR (95% CI)	*P*
**Control**	1.00 (ref.)		1.00 (ref.)	
**Insomnia**	1.59 (1.28-1.97)	< 0.001	1.38 (1.06-1.79)	0.02

HTN = hypertension; CVD = cardiovascular disease; AHR = adjusted hazard ratio; CI = confidence interval; ref. = reference value.

Discussion

This is the largest cohort study focused on the association between insomnia and new-onset asthma, and the first one on an Asian population. We found that insomnia patients who sought medical assistance had a significant risk for developing asthma within 4 years of asking for treatment. In this study, the incidence of asthma in control group was 10.57 per 10,000 person-years. This finding is similar to the most recent epidemiology study[25] in Taiwan that the incidence of asthma was 9.8 per 10,000 person-year. In contrast, the insomnia group had significant higher incidence of asthma – 22.01 per 10,000 person-year than the control group and the general population in previous study.[25] Several studies have reported linkages between insomnia and chronic illnesses, including asthma. A cross-sectional study[19] of 3283 adults showed a higher prevalence of insomnia in those with asthma (AOR 1.6; 95% CI: 1.3-2.0). A 5-year prospective study[26] of 2316 middle-aged adults reported that patients with insomnia at baseline had a higher incidence of asthma (AOR = 17.9; 95% CI: 2.28-140) than did patients without insomnia. But no causal relationship between insomnia and asthma was supported in these two studies. Unlike those two studies, our study excluded patients who had previously been diagnosed with asthma, and we followed our patients for 4 years, which provided evidence that insomnia is a risk factor for developing asthma.

Sivertsen et al.[20] reported that insomnia was a significant risk factor for incidence of

For peer review only

asthma in an 11-year large population-based prospective cohort study consisted of 24715 participants (OR = 1.47; 95% CI: 1.16-1.86). Brumpton et al.[27] also found that insomnia symptoms were associated with increased risk of incident asthma in the same population-based which consisted of 17927 participants (three insomnia symptoms, OR = 1.70; 95% CI: 1.37–2.11). However, they used questionnaires to define insomnia and provided no information about the severity or duration of their participants' poor sleep. The diagnoses of asthma and comorbidities were based on self-reports instead of physician-reported diagnoses. Their sample size was smaller than was ours, and the confounding factors adjusted for in their analysis did not include specific asthma or insomnia-related comorbidities such as atopy. In contrast, we used ICD-9 CM codes from Taiwan's LHID2000, which indicated the patients' physician-diagnosed illnesses, including insomnia, asthma, and all comorbidities. Besides, we believed that the insomniacs in our cohort suffered from more severe sleep-related symptoms that needed medical assistance. Our findings were statistically significant after our analyses had been fully adjusted for the most common confounding factors related to asthma and insomnia.

Although our findings and those of other studies[17,18,20] indicate a significant association between insomnia and asthma, the pathophysiology of insomnia-related asthma is still unclear. Nonetheless, the common inflammatory pathway between insomnia and asthma should be considered, and several mechanisms have been proposed to explain the potential relationship between insomnia and asthma. First, poor sleep increases Interleukin-6 (IL-6) production, and this response lasts until daytime.[28,29] Patients with stable and with acute asthma had significantly higher IL-6 production levels in serum, sputum, and bronchoalveolar lavage fluid than did healthy controls.[30,31] IL-6 production in the airway promotes allergic airway inflammation in mice. In contrast, using IL-6 knockout mice in the same model showed significantly less mucus secretion.[32,33] Therefore, insomnia might contribute to inducing IL-6

For peer review only

production and that might exacerbate airway hypersensitivity. Second, NF-κB can be induced by sleep loss.[34] Prolonged airway epithelial NF-κB activation has been reported in patients with asthma. Even temporal NF-κB activation in the airway epithelium is sufficient to induce airway hyperresponsiveness in mice.[35] Thus, the higher incidence of asthma in insomnia patients might be the result of temporal or persistent NF-κB activation induced by sleep loss. Third, insomnia is related to a reduction in interferon (IFN)-γ.[36] IFN-γ production, which inhibits airway epithelial inflammation, is lower in asthma patients than in healthy controls.[37,38] These studies suggest that IFN-γ plays a significant role between insomnia and asthma.

The strengths of our study are its prospective cohort design and that we used the LHID2000, a subset of Taiwan's National Health Insurance Research Database (NHIRD), which contains physician-provided clinical diagnoses instead of illnesses self-reported by patients. The LHID2000 reflects the real-world situation in Taiwan and is more generalizable than are hospital-based or city-based databases. The large sample size and long-term follow-up also provide considerable statistical power.

Our study also has some limitations. First, the NHIRD does not include results of pulmonary function tests or blood tests for inflammatory cytokines, severity levels or the actual duration of insomnia, some important confounding factors, such as exposure to house dust mites, smoking habits, body mass index, family history, or medical compliance. Second, we did not assess the association between the medication for insomnia and asthma. However, it would be interesting to note if there were any differences in the development of asthma in those insomnia patients who were treated versus those who were not. Further study is warranted to investigate the drug effect. Third, some patients might have minor asthmatic symptoms and have not been diagnosed yet before insomnia. Forth, sampling bias, such as a higher number of individuals likely to seek medical care among insomnia group than control

For peer review only

group is possible and it may confound the analysis. Finally, because the NHIRD does not contain patient information before 1996, some of our patients might have been misclassified if they were diagnosed with asthma or insomnia before that year.

Conclusion

We found that insomnia patients who required medical assistance had a higher risk for developing new-onset asthma. Proper treatments for insomnia patients might help prevent the progress of airway inflammation. Additional study is needed to identify the actual mechanism that connects insomnia and asthma.

Author contributions:

YCL, CCL, and KCC designed the study, interpreted the data and drafted, and revised the article. CCC, CMC, and SRC contributed to interpreting the data and revising the article. CHH and SFW contributed to the statistical analysis. KCC critically reviewed and revised the article. All of the authors read and agreed with the final version of the manuscript.

Data sharing statement:

The data on the study population that were obtained from the NHIRD (https://nhird.nhri.org.tw/en/) are maintained in the NHRI (http://nhird.nhri.org.tw/). The NHIRD is limited for research purposes only. Applicants must follow the Computer-Processed Personal Data Protection Law (http://www.winklerpartners.com/?p=987) and related regulations of National Health Insurance Administration. All applications are reviewed for approval of data release. Interested researchers may submit queries related to data access to nhird@nhri.org.tw

For peer review only

Figure Legends

Figure 1.The flowchart of study subjects selection

Figure 2. The probability of developing asthma in insomnia and control group

For peer review only

References

1. American Psychiatric Association. Diagnostic and statistical manual of mental disorders, fifth edition. Arlington, VA: Author, 2013.
2. Kao CC, Huang CJ, Wang MY, Tsai PS. Insomnia: prevalence and its impact on excessive daytime sleepiness and psychological well-being in the adult Taiwanese population. Qual Life Res 2008;17:1073-1080.
3. Cunnington D, Junge MF, Fernando AT. Insomnia: prevalence, consequences and effective treatment. Med J Aust 2013;199:S36-S40.
4. Sivertsen B, Krokstad S, Øverland S, Mykletun A. The epidemiology of insomnia: associations with physical and mental health. The HUNT-2 study. J Psychosom Res 2009;67:109-116.
5. Walsh JK. Clinical and socioeconomic correlates of insomnia. J Clin Psychiatry 2004;65 Suppl 8:13-19.
6. Roane BM, Taylor DJ. Adolescent insomnia as a risk factor for early adult depression and substance abuse. Sleep 2008;31:1351-356.
7. Neckelmann D, Mykletun A, Dahl AA. Chronic insomnia as a risk factor for developing anxiety and depression. Sleep 2007;30:873-880.
8. Jaussent I, Bouyer J, Ancelin M-L, Akbaraly T, Pérès K, Ritchie K, et al. Insomnia and daytime sleepiness are risk factors for depressive symptoms in the elderly. Sleep 2011;34:1103-1110.
9. Chien KL, Chen PC, Hsu HC, Su TC, Sung FC, Chen MF, et al. Habitual sleep duration and insomnia and the risk of cardiovascular events and all-cause death: report from a community-based cohort. Sleep 2010;33:177-184.
10. Kronholm E, Laatikainen T, Peltonen M, Sippola R, Partonen T. Self-reported sleep duration, all-cause mortality, cardiovascular mortality and morbidity in Finland. Sleep

For peer review only

Med 2011;12:215-221.

11. Ozminkowski RJ, Wang S, Walsh JK. The direct and indirect costs of untreated insomnia in adults in the United States. Sleep 2007;30:263-273.
12. Vgontzas AN, Liao D, Pejovic S, Calhoun S, Karataraki M, Basta M, et al. Insomnia with short sleep duration and mortality: the Penn State cohort. Sleep 2010;33:1159-1164.
13. Gangwisch JE, Heymsfiel SB, Boden-Albala B, Buijs RM, Kreier F, Pickering TG, et al. Sleep duration as a risk factor for diabetes incidence in a large US sample. Sleep 2007;30:1667-1673.
14. Gangwisch JE, Malaspina D, Boden-Albala B, Heymsfiel SB. Inadequate sleep as a risk factor for obesity: analyses of the NHANES I. Sleep 2005;28:1289-1296.
15. Smolensky MH, Milia LD, Ohayon MM, Philip P. Sleep disorders, medical conditions, and road accident risk. Accid Anal Prev 2011;43:533-548.
16. Global Initiative for Asthma. 2015 GINA Report, Global Strategy for Asthma Management and Prevention: p. 2. Available from: http://ginasthma.org/gina-report-global-strategy-for-asthma-management-and-prevention/
17. Meltzer LJ, Ullrich M, Szefler SJ. Sleep duration, sleep hygiene, and insomnia in adolescents with asthma. J Allergy Clin Immunol Pract 2014;2:562-569.
18. Sundbom F, Lindberg E, Bjerg A, Forsberg B, Franklin K, Gunnbjörnsdottir M, et al. Asthma symptoms and nasal congestion as independent risk factors for insomnia in a general population: results from the GA(2)LEN survey. Allergy 2013;68:213-219.
19. Budhiraja R, Roth T, Hudgel DW, Budhiraja P, Drake CL. Prevalence and polysomnographic correlates of insomnia comorbid with medical disorders. Sleep 2011;34:859-867.
20. Sivertsen B, Lallukka T, Salo P, Pallesen S, Hysing M, Krokstad S, et al. Insomnia as a

For peer review only

risk factor for ill health: results from the large population-based prospective HUNT Study in Norway. J Sleep Res 2014;23:124-132.

21. Chen PJ, Huang CLC, Weng SF, Wu MP, Ho CH, Wang JJ, et al. Relapse insomnia increases greater risk of anxiety and depression: evidence from a population-based 4-year cohort study. Sleep Med 2017;38:122-129

22. Huang CLC, Weng SF, Wang JJ, Hsu YW, Wu MP. Risks of treated insomnia, anxiety, and depression in health care-seeking physicians: a nationwide population-based study. Medicine 2015;94:e1323.

23. Chuang YC, Weng SF, Hsu YW, Huang CLC, Wu MP. Increased risks of healthcare-seeking behaviors of anxiety, depression and insomnia among patients with bladder pain syndrome/interstitial cystitis: a nationwide population-based study. Int Urol Nephrol 2015;47:275-281.

24. Wu MP, Lin HJ, Weng SF, Ho CH, Wang JJ, Hsu YW. Insomnia Subtypes and the Subsequent Risks of Stroke. Stroke 2014;45:1349-1354.

25. Wu TJ, Wu CF, Lee YL, Hsiue TR, Guo YL. Asthma incidence, remission, relapse and persistence: a population-based study in southern Taiwan. Respir Res 2014;15:135.

26. Zhang J, Lama SP, Li SX, Yu MWM, Li AM, Ma RCW, et al. Long-term outcomes and predictors of chronic insomnia: a prospective study in Hong Kong Chinese adults. Sleep Med 2012;13:455-462.

27. Brumpton B, Mai XM, Langhammer A, Laugsand LE, Janszky I, Strand LB. Prospective study of insomnia and incident asthma in adults: the HUNT study. European Respiratory Journal Feb 2017;49:1601327

28. Burgos I, Richter L, Klein T, Fiebich B, Feige B, Lieb K, et al. Increased nocturnal interleukin-6 excretion in patients with primary insomnia: a pilot study. Brain Behav Immun 2006;20:246-253.

For peer review only

29. Voderholzer U, Fiebich BL, Dersch R, Feige B, Piosczyk H, Kopasz M, et al. Effects of sleep deprivation on nocturnal cytokine concentrations in depressed patients and healthy control subjects. J Neuropsychiatry Clin Neurosci 2012;24:354-366.

30. Neveu WA, Allard JL, Raymond DM, Bourassa LM, Burns SM, Bunn JY, et al. Elevation of IL-6 in the allergic asthmatic airway is independent of inflammation but associates with loss of central airway function. Respir Res 2010;11:28.

31. Yokoyama A, Kohno N, Fujino S, Hamada H, Inoue Y, Fujioka S, et al. Circulating interleukin-6 levels in patients with bronchial asthma. Am J Respir Crlt Care Med 1995;151:1354-1358.

32. Rincon M, Irvin CG. Role of IL-6 in asthma and other inflammatory pulmonary diseases. Int J Biol Sci 2012;8:1281-1290.

33. Neveu WA, Allard JB, Dienz O, Wargo MJ, Ciliberto G, Whittaker LA, et al. IL-6 is required for airway mucus production induced by inhaled fungal allergens. J Immunol 2009;183:1732-1738.

34. Irwin MR, Wang M, Ribeiro D, Cho HJ, Olmstead R, Breen EC, et al. Sleep loss activates cellular inflammatory signaling. Biol Psychiatry 2008;64:538-540.

35. Ather JL, Hodgkins SR, Janssen-Heininger YMW, Poynter ME. Airway epithelial NF-kappa B activation promotes allergic sensitization to an innocuous inhaled antigen. Am J Respir Cell Mol Biol 2011;44:631-638.

36. Sakami S, Ishikawa T, Kawakami N, Haratani T, Fukui A, Kobayashi F, et al. Coemergence of insomnia and a shift in the Th1/Th2 balance toward Th2 dominance. Neuroimmunomodulation 2002-2003;10:337-343.

37. Lama M, Chatterjee M, Nayakc CR, Chaudhuri TK. Increased interleukin-4 and decreased interferon-gamma levels in serum of children with asthma. Cytokine 2011;55:335-338.

For peer review only

38. Mitchell C, Provost K, Niu N, Homer R, Cohn L. IFN-gamma acts on the airway epithelium to inhibit local and systemic pathology in allergic airway disease. J Immunol 2011;187:3815-3820.

For peer review only

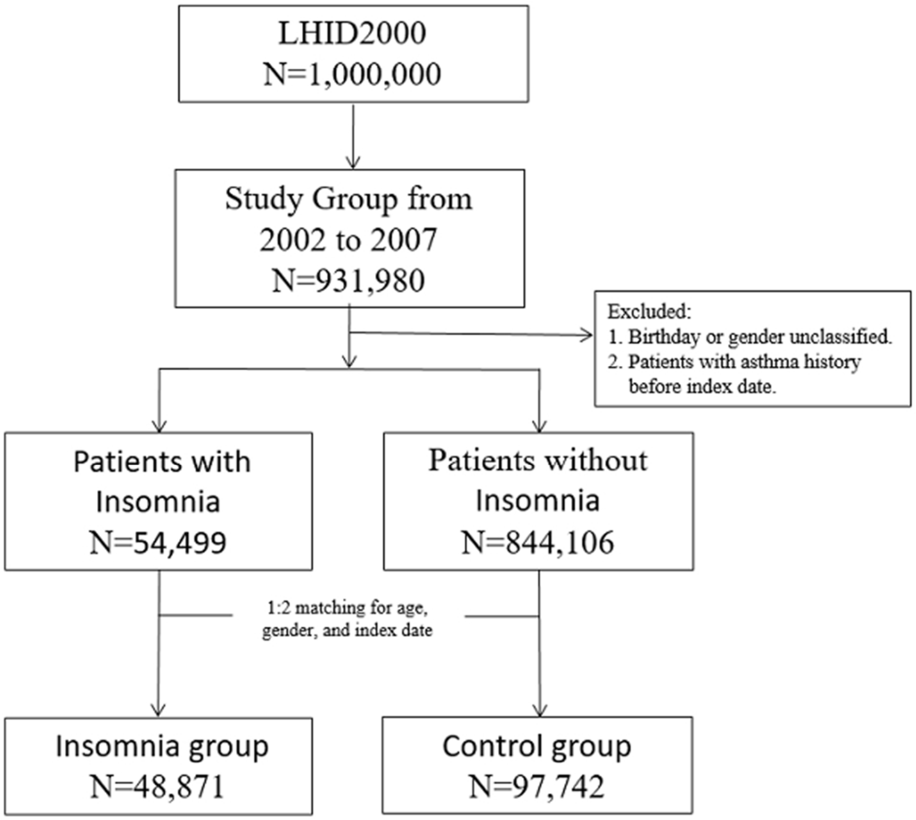

Figure 1.The flowchart of study subjects selection

86x77mm (300 x 300 DPI)

only

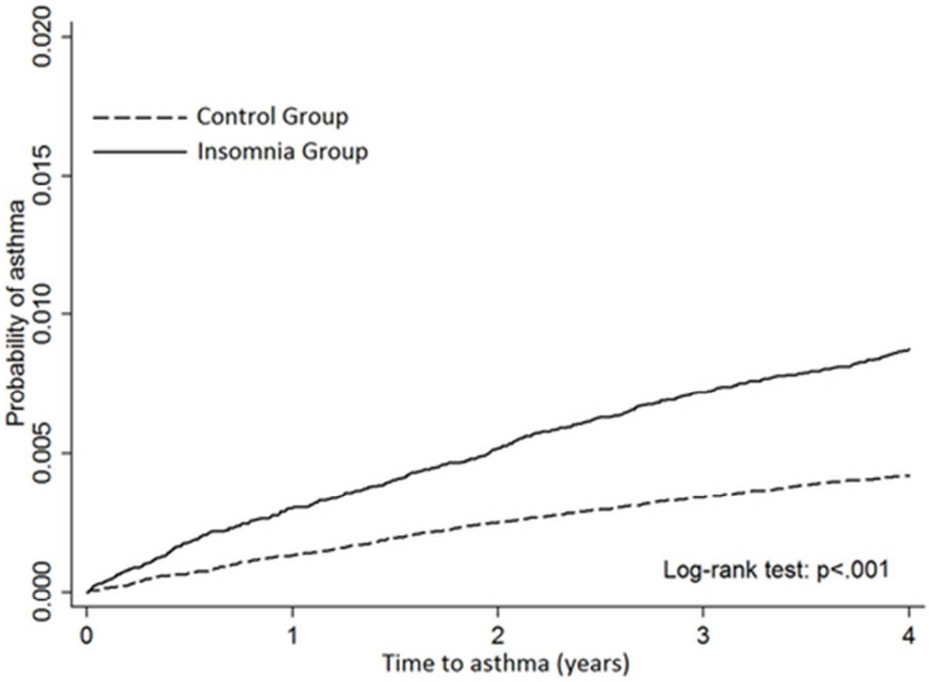

Figure 2. The probability of developing asthma in insomnia and control group

45x32mm (300 x 300 DPI)

For peer review only

STROBE 2007 (v4) Statement—Checklist of items that should be included in reports of *cohort studies*

Section/Topic	Item #	Recommendation	Reported on page #
**Title and abstract**	1	(*a*) Indicate the study's design with a commonly used term in the title or the abstract	1,2
		(*b*) Provide in the abstract an informative and balanced summary of what was done and what was found	2
**Introduction**			
Background/rationale	2	Explain the scientific background and rationale for the investigation being reported	4
Objectives	3	State specific objectives, including any prespecifid hypotheses	4
**Methods**			
Study design	4	Present key elements of study design early in the paper	4-6
Setting	5	Describe the setting, locations, and relevant dates, including periods of recruitment, exposure, follow-up, and data collection	4-6
Participants	6	(*a*) Give the eligibility criteria, and the sources and methods of selection of participants. Describe methods of follow-up	4-6
		(*b*) For matched studies, give matching criteria and number of exposed and unexposed	4-6
Variables	7	Clearly define all outcomes, exposures, predictors, potential confounders, and effect modifiers. Give diagnostic criteria, if applicable	4-6
Data sources/ measurement	8*	For each variable of interest, give sources of data and details of methods of assessment (measurement). Describe comparability of assessment methods if there is more than one group	4-6
Bias	9	Describe any efforts to address potential sources of bias	4-6
Study size	10	Explain how the study size was arrived at	4-6
Quantitative variables	11	Explain how quantitative variables were handled in the analyses. If applicable, describe which groupings were chosen and why	4-6
Statistical methods	12	(*a*) Describe all statistical methods, including those used to control for confounding	4-6
		(*b*) Describe any methods used to examine subgroups and interactions	4-6
		(*c*) Explain how missing data were addressed	4-6
		(*d*) If applicable, explain how loss to follow-up was addressed	4-6
		(*e*) Describe any sensitivity analyses	4-6
**Results**			

For peer review only

Participants	13*	(a) Report numbers of individuals at each stage of study—eg numbers potentially eligible, examined for eligibility, confirmed eligible, included in the study, completing follow-up, and analysed	6-12
		(b) Give reasons for non-participation at each stage	6-12
		(c) Consider use of a flow diagram	6-12
Descriptive data	14*	(a) Give characteristics of study participants (eg demographic, clinical, social) and information on exposures and potential confounders	6-12
		(b) Indicate number of participants with missing data for each variable of interest	6-12
		(c) Summarise follow-up time (eg, average and total amount)	6-12
Outcome data	15*	Report numbers of outcome events or summary measures over time	6-12
Main results	16	(*a*) Give unadjusted estimates and, if applicable, confounder-adjusted estimates and their precision (eg, 95% confidence interval). Make clear which confounders were adjusted for and why they were included	6-12
		(*b*) Report category boundaries when continuous variables were categorized	6-12
		(*c*) If relevant, consider translating estimates of relative risk into absolute risk for a meaningful time period	6-12
Other analyses	17	Report other analyses done—eg analyses of subgroups and interactions, and sensitivity analyses	6-12
**Discussion**			
Key results	18	Summarise key results with reference to study objectives	12
**Limitations**			
Interpretation	20	Give a cautious overall interpretation of results considering objectives, limitations, multiplicity of analyses, results from similar studies, and other relevant evidence	13-14
Generalisability	21	Discuss the generalisability (external validity) of the study results	14
**Other information**			
Funding	22	Give the source of funding and the role of the funders for the present study and, if applicable, for the original study on which the present article is based	15

*Give information separately for cases and controls in case-control studies and, if applicable, for exposed and unexposed groups in cohort and cross-sectional studies.

Note: An Explanation and Elaboration article discusses each checklist item and gives methodological background and published examples of transparent reporting. The STROBE checklist is best used in conjunction with this article (freely available on the Web sites of PLoS Medicine at http://www.plosmedicine.org/, Annals of Internal Medicine at http://www.annals.org/, and Epidemiology at http://www.epidem.com/). Information on the STROBE Initiative is available at www.strobe-statement.org.

For peer review only
